# AspFlex: Molecular Tools to Study Gene Expression and Regulation in *Acinetobacter baumannii*

**DOI:** 10.1021/acssynbio.3c00167

**Published:** 2023-08-16

**Authors:** Merlin Brychcy, Alexis Kokodynski, Devin Lloyd, Veronica G. Godoy

**Affiliations:** Biology Department, Northeastern University, Boston, Massachusetts 02115, United States

**Keywords:** *Acinetobacter baumannii*, MoClo, Golden Gate, gene regulation, CRISPRi, dCas9

## Abstract

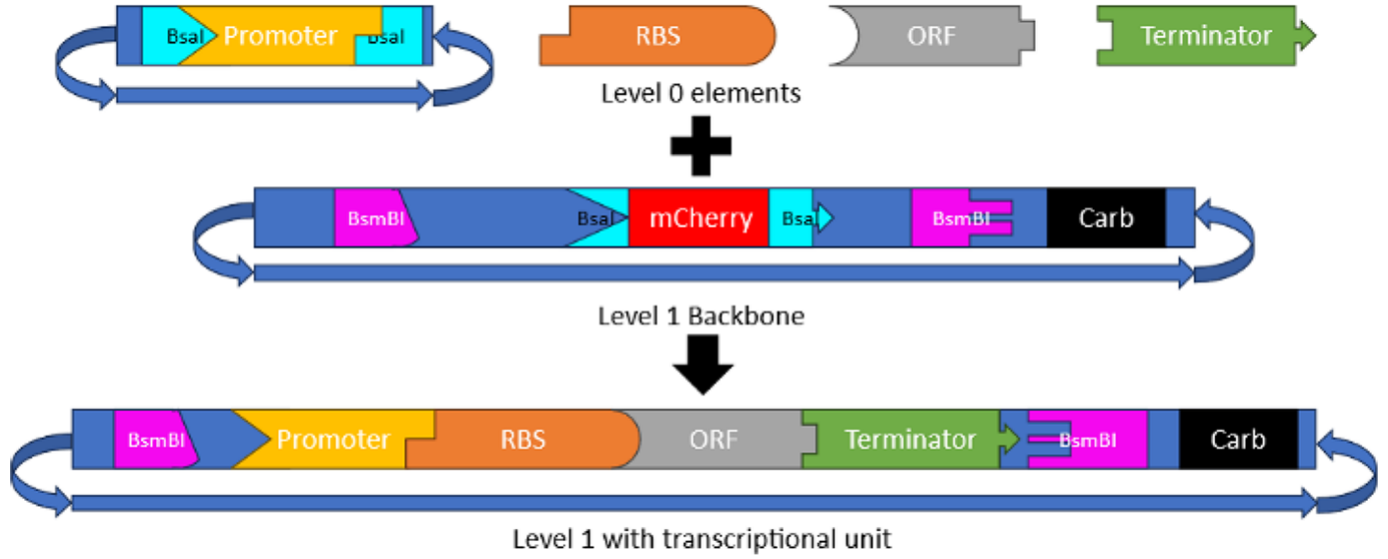

*Acinetobacter baumannii* is a Gram-negative nosocomial opportunistic pathogen frequently found in hospital settings, causing high incidence of in-hospital infections. It belongs to the ESKAPE group of pathogens (the “A” stands for *A. baumannii*), which are known to easily develop antibiotic resistances. It is crucial to create a molecular toolkit to investigate its basic biology, such as gene regulation. Despite *A. baumannii* having been a threat for almost two decades, an efficient and high-throughput plasmid system that can replicate in *A. baumannii* has not yet been developed. This study adapts an existing toolkit for *Escherichia coli* to meet *A. baumannii*’s unique requirements and expands it by constructing a plasmid-based CRISPR interference (CRISPRi) system to generate gene knockdowns in *A. baumannii*.

## Introduction

*Acinetobacter baumannii* is a worldwide nosocomial opportunistic pathogen with high morbidity and mortality.^1^*A. baumannii* infections have risen sharply during the last 20 years; the CDC classifies it as a serious threat level, accounting for 12,000 infections every year.^2^*A. baumannii*’s main strategies to be such a successful pathogen rely on acquisition of antibiotic resistances and ability to survive desiccation by existing in multicellular communities or biofilms.^3^ Over half of the infections caused by *A. baumannii* are by multidrug-resistant strains.^4^

Having a diverse array of molecular tools is essential to understand the mechanisms governing basic molecular processes in *A. baumannii* and to discover innovative strategies to eradicate it. Although tools and expression plasmids are already accessible,^5,6^ such as plasmids for allelic exchange^7^ or transposon insertion^8^ or genome editing,^9,10^ they depend on conventional restriction enzyme cloning, making them tedious, time-consuming, and inefficient, and the cloning of several inserts is cumbersome. More sophisticated genetic instruments like CRISPR interference (CRISPRi)^11^ are still in the early stages of development.^12,13^ CRISPRi offers a valuable way to investigate essential genes in greater depth. This is significant considering the large number of genes of unknown function present in the *A. baumannii* genome. The main advantage of Golden Gate cloning is the use of Type IIS restriction enzymes that cleave outside of their recognition site. This enables the creation of custom overhangs for directional cloning as well as combining digestion and ligation reactions in a single tube, resulting in a significantly greater cloning efficiency.^14,15^ This easy and efficient system was a prime platform to clone gene editing fragments, including TALEN^16^ and CRISPR/Cas9 sgRNAs.^17,18^ Golden Gate cloning systems are well-established for *E. coli*,^19,20^ but they are currently unavailable for *A. baumannii*.

The aim of this study was to devise a Golden Gate-based plasmid system for *A. baumannii*, which will facilitate the study of any gene of interest into plasmids. We report the creation of a plasmid kit (AddgeneID: 1000000217) with the possibility to express up to 20 transcriptional units at once by adjusting the already established EcoFlex system^19^ to *A. baumannii*’s needs. We demonstrate here its use by creating an *A. baumannii* plasmid-based CRISPRi gene system.

## Results

### Construction of AspFlex, a Golden Gate Cloning System for *A. baumannii*

**Figure 1:**
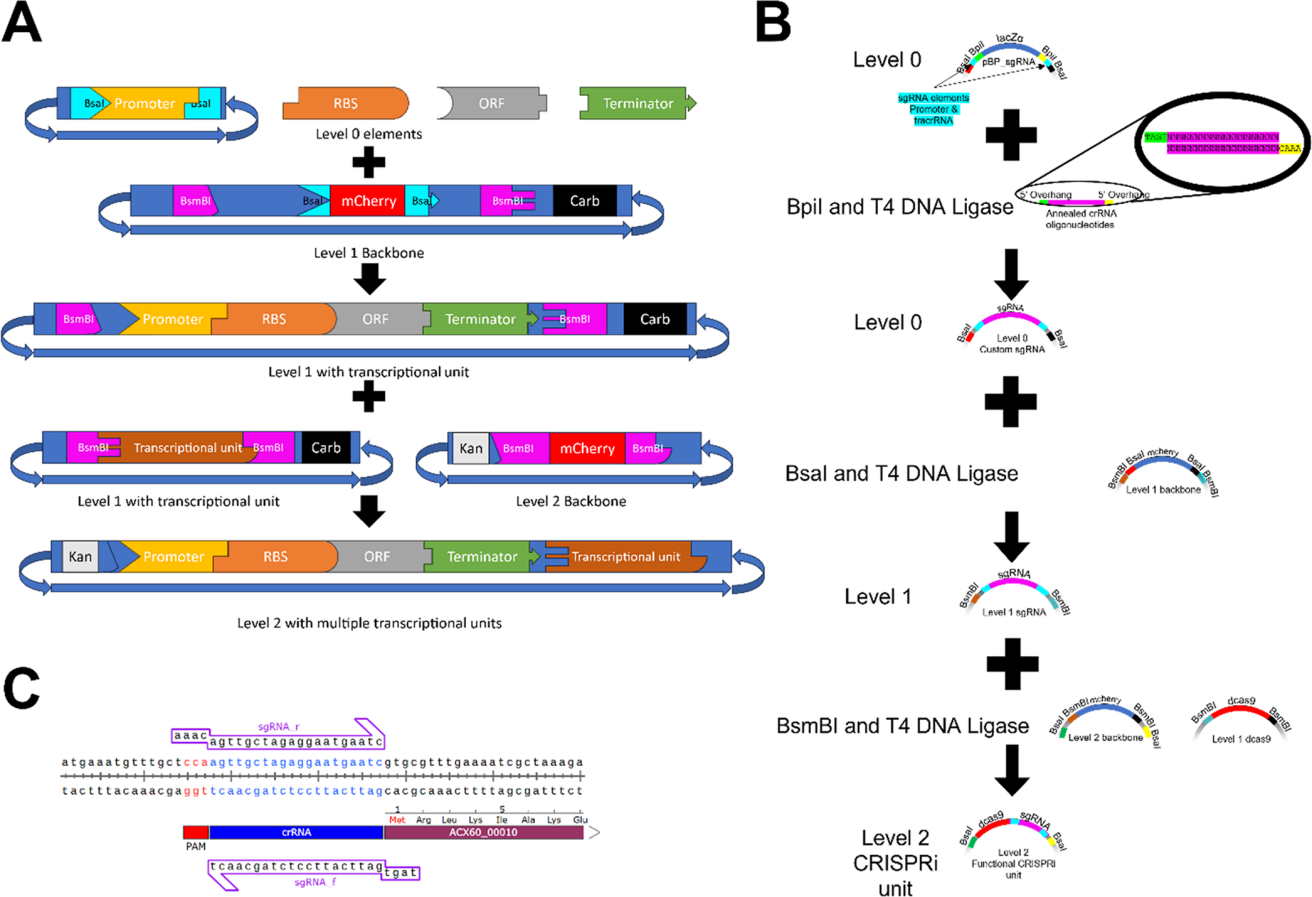
Schematic of AspFlex cloning approaches. (A) Cloning of a transcriptional unit (TU). Level 0 shows the elements used to form a TU either in a plasmid or as a PCR fragment flanked by BsaI recognition sites generating unique overhangs. BsaI and all other Type IIS restriction enzymes display endonuclease activity outside of their recognition site, allowing the design of unique overhangs and therefore the design of a puzzle system for the fragments, as visualized here. A functional single TU is formed, combined in a Golden Gate reaction with BsaI and T4 DNA ligase. Multiple TU fragments (or level 1 plasmids) can then be combined to form level 2 plasmids using BsmBI and T4 DNA ligase. Complementary overhangs are indicated by their shapes. Each level contains a unique resistance marker, allowing the selection via both the antibiotic and through screening of white colonies due to the lack of *mcherry* present in the cloning site. (B) Diagram for the construction of a CRISPRi system using AspFlex. First, pBP_sgRNA, two complementary oligonucleotides, BpiI and T4 DNA ligase, form a single level 0 plasmid containing a functional sgRNA unit. The fragment present is then cloned into a level 1 backbone using BsaI and T4 DNA ligase, forming a level 1 plasmid containing a single sgRNA. Multiple level 1 sgRNA fragments can then be used to create a functional CRISPRi in a level 2 plasmid by combination with a level 1 fragment containing *dCas9* in a level 2 backbone. Complementary overhangs are indicated via the name of the restriction enzyme generating them and their color. (C) Example of the design of an sgRNA for CRISPRi. The crRNA is shown in blue, and the PAM is in red. The gene suppressed by CRISPRi is shown in plum, and the two complementary oligonucleotides are shown in purple. This figure was generated with Snapgene (http://snapgene.com).

The EcoFlex system^19^ is organized in a standard modular cloning (MoClo) fashion based on multiple levels of organization. Level 0 plasmids contain elements, such as promoters, terminators, or open reading frames. These are used to build a transcriptional unit (TU) in level 1 plasmids, which allows for the expression of a single TU by combining level 0 elements. Subsequently, level 2 plasmids allow the expression of multiple TUs by combining multiple level 1 plasmids, and so on (Figure 1A).

To adapt EcoFlex^19^ to *A. baumannii*, we introduced the replication elements from a plasmid from *A. calcoaceticus*,^21^ pWH1266, which is known to replicate in *A. baumannii*(6) and in other *Acinetobacter* spp.^22^ The replication elements were amplified using primers ori_ab_psti_f and ori_ab_psti_r (Supplemental Table 1) and introduced into the level 1–3 plasmids using *Pst*I-HF (New England Biolabs) followed by ligation with T4 DNA ligase (New England Biolabs). Since *A. baumannii* is resistant to chloramphenicol,^23−25^ the antibiotic marker present in the EcoFlex level 2 plasmids, the *cat* gene encoding a chloramphenicol acetyltransferase resistance cassette was digested with EcoRV-HF (New England Biolabs) and interrupted with a blunt-ended fragment containing the *kanR* gene fragment with its own promoter. Lastly, to generate plasmids usable in multidrug-resistant *A. baumannii* strains, we generated a version of each level plasmid containing an additional tellurite resistance cassette (TelR). Details of the TelR plasmid construction can be found in the Supporting Information.

The result of these manipulations is a set of level 1 (pMB1-A through pMB1-E, AddgeneIDs 190114–190119), level 2 (pMB2-A through pMB2-D, AddgeneIDs 190120–190125), and level 3 (pMB3-A and pMB3-B, AddgeneIDs 190126 and 120127) plasmids as well as a set of the same plasmids containing the additional tellurite resistance cassette (AddgeneIDs 204979–204992). To test whether plasmids would stably replicate and be maintained in *A. baumannii* ATCC17978, we used a pMB1-A derivative, a level 1 plasmid strongly expressing *egfp*(26), and introduced it by transformation into *A. baumannii*. We expected that *A. baumannii* cells in which the plasmid was successfully replicated would be both resistant to the plasmid marker and bright-green-fluorescent for multiple generations. This is indeed what we found (Supplemental Figure 1). Additionally, we sequenced the full plasmids extracted from *A. baumannii* on day 1 and day 5 to assess whether any recombination or mutation events had occurred. We found none with and without the addition of carbenicillin as the selecting agent (see the Supporting Information for the full sequence alignment).

### AspFlex and CRISPRi

To create the plasmid-based CRISPRi gene system, we first produced the dCas9 plasmid. To do so, elements such as an anhydrotetracycline (ATc)-inducible promoter (pTet) with the pET-RBS ribosomal binding site (AddgeneID 72981) to tightly regulate the expression of the catalytically deficient Cas9 gene from pdCas9 bacteria (AddgeneID 44249)^11^ and the terminator Bba_B0012 (AddgeneID 190129) were moved onto pMB1-A. The catalytically deficient Cas9 gene,^11^*dCas9*, was amplified using primers pdCas9_new_r and pdCas9_new_f (Supplemental Table 3). The resulting fragment was cloned into a level 0 plasmid. However, to move this fragment to the level 1 pMB1-A plasmid on AspFlex, a BsmBI site, a Type IIS restriction enzyme required for cloning in level 2 plasmids, needed to be removed from the dCas9 fragment. To achieve this, site-directed mutagenesis was carried out with primers pBP_cas9_mut_r and pBP_cas9_mut_f (Supplemental Table 3).

Another level 0 plasmid carrying the guide RNAs (sgRNAs) also needed to be implemented. For easy selection and Golden Gate-compatible cloning of sgRNAs, we designed a custom fragment (Figure 1B). Here, a *lacZ* alpha fragment for blue-white screening is flanked on one side by the constitutively expressed strong promoter J23119^12^ and on the other by sequences encoding the tracrRNA. A simple reaction with two complementary oligonucleotides including the Bpi-HF (NEB) sequence plus two distinct overhangs was used to generate directional cloning of a functional sgRNA. Thus, the AspFlex plasmid, pBP_sgRNA, (AddgeneID 190128) allows easy cloning of constitutive expression of sgRNAs (Figure 1B). Screening of white positive colonies on X-Gal plates is carried out by PCR with flanking primers. We determined cloning efficiency by counting white colonies on X-Gal that contained the expected construct and found this to be excellent: 100% of white colonies analyzed were correct (Supplemental Figure 2).

**Figure 2:**
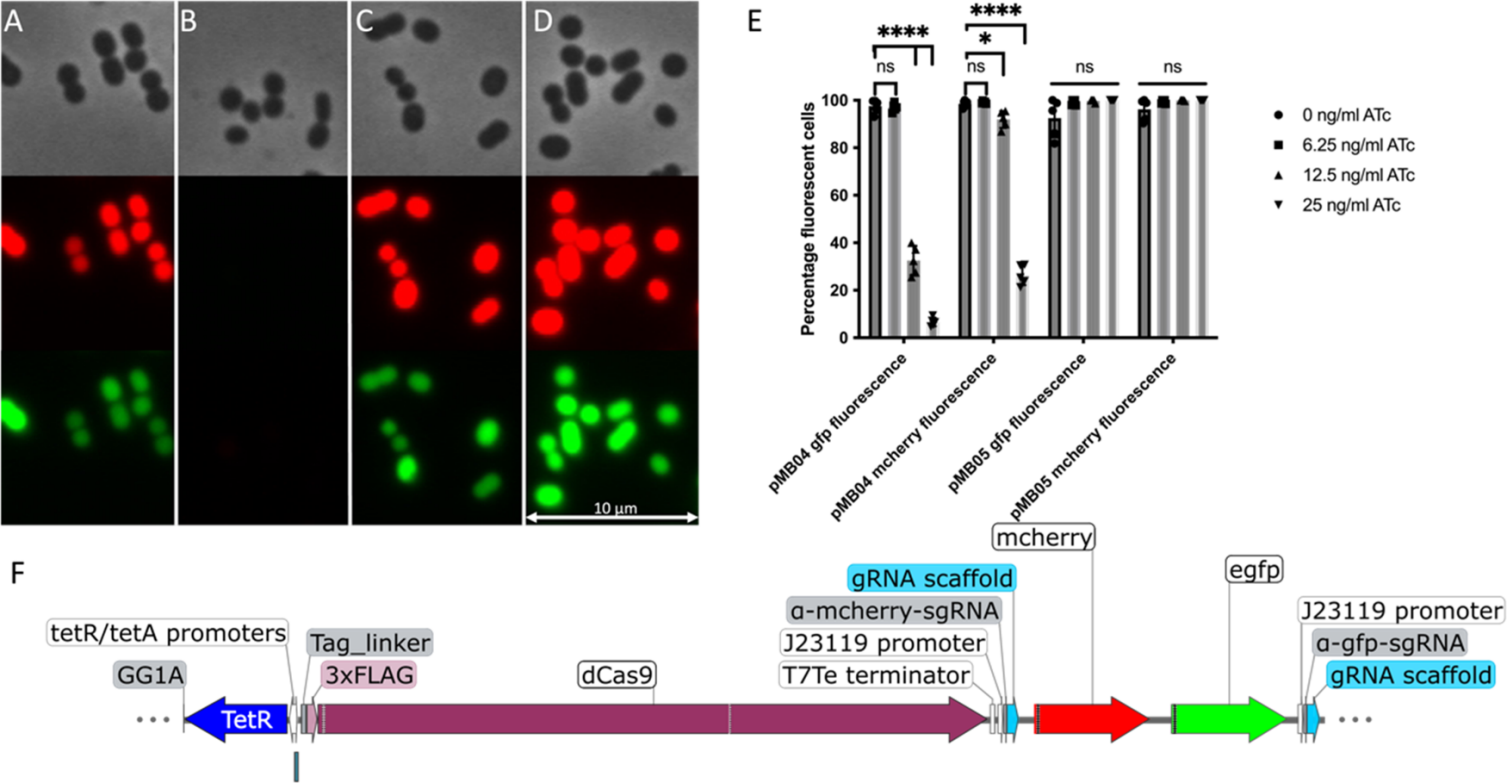
Fluorescence is suppressed by CRISPRi. (A–D) Phase-contrast microscope images (top), red fluorescence channel microscope image (middle), and green fluorescence channel image (bottom). Data were collected by subculturing saturated cells with ATc for 3 h, with subsequent microscopy analysis and quantification of fluorescence with ImageJ^27^ and Fiji.^28^ (A) *A. baumannii* containing pMB04 construct (sgRNAs targeting fluorescence genes) with no ATc, the dCas9 gene inducing agent. (B) *A. baumannii* containing pMB04 construct with 25 ng/mL ATc. (C) *A. baumannii* with pMB05 construct (nonsense sgRNAs) with no ATc. (D) *A. baumannii* cells with pMB05 with 25 ng/mL ATc. (E) Quantification of percentage of fluorescent cells at different concentrations of inducing agent ATc. The percentage of fluorescent cells is significantly reduced only when sgRNAs targeting the fluorescence genes are present and not observed when nonsense sgRNAs are used. (F) Insert of plasmid pMB04. The insert contains an ATc-inducible dCas9 (blue and plum), two fluorescence genes (*egfp* and *mcherry*; green and red), and sgRNAs targeting the two genes (gray and teal). The binding site of the sgRNAs on the appropriate fluorescence gene is marked with a black line within the fluorescence gene. This figure was generated with SnapGene (http://www.snapgene.com).

To test the CRISPRi system shown in Figure 1B in *A. baumannii*, two level 2 proof-of-principle plasmids were constructed. One of these plasmids, pMB04, contains an ATc-inducible *dCas9* gene, a strongly expressed *egfp* and *mcherry* gene, and constitutively expressed sgRNAs targeting the fluorescent protein genes (Figure 2F). The other, pMB05, contains the same fluorescent genes and no-targeting nonsense sgRNAs (see the Supporting Information for sequences of both plasmids). We tested the fluorescence intensity of *A. baumannii* ATCC17978 strains with either plasmid with different concentrations of the *dCas9* inducer ATc and with no ATc (Figure 2). We found that in ATc-treated cells with pMB04, the plasmid with the fluorescent proteins targeting sgRNAs, the fluorescence signal is significantly repressed, but not with pMB05 carrying the nonsense nontargeting sgRNA (see Figure 2A–D). We found that increasing the concentration of ATc intensified this effect (Figure 2E). The data indicate that the CRISPRi platform is effective in *A. baumannii*. Furthermore, the platform also allows *dCas9* expression with different promoters, constitutive or inducible (for example, pBAD, included in the set; AddgeneID 190132).

The results from these experiments demonstrate the effective use of a MoClo-based CRISPRi plasmid system in *A. baumannii*, providing opportunities for further research on essential genes and gene regulation. By using this system, it is possible to precisely control the expression of a regulator and evaluate its impact. This approach presents a wide range of possibilities for studying *A. baumannii*.

In summary, AspFlex is shown to be an ideal, efficient, fast, and easy method to construct a wide range of genetic tools such as transcriptional reporters or protein fusions. We demonstrate here its use by setting up a plasmid-based CRISPRi system that allows the regulation of multiple transcripts at once, providing a wide range of possibilities to study the functionality of gene pathways in *A. baumannii*.

## Experimental Procedures

Detailed protocols for all methods used in this report are given in the Supporting Information.

### Design of sgRNA Oligos and Cloning in Level 0 Plasmids

To create a CRISPRi sgRNA, appropriate sites in the promoter region/early open reading frame of the fluorescence genes containing an NGG PAM site were identified. Two complementary oligonucleotides of 20–24 bp length were designed with the one on the same strand as the NGG having a TAGT 5′ and the complementary oligonucleotide having a AAAC 5′ overhang (see Figure 1C). The two oligonucleotides were annealed in ligase buffer by heating to 95 °C for 5 min and subsequent incubation at 22 °C for 20 min. A Golden Gate reaction using the Type IIS restriction enzyme BpiI, T4-DNA ligase, pBP_sgRNA (AddgeneID 190128), and the annealed oligos was then used to clone the custom sgRNA. Positive clones were screened using blue-white color formation in plates with X-Gal (40 μg/mL). The fragments in the plasmid can be used in subsequent Golden Gate reactions to construct a level 1 MoClo plasmid with a level 1 backbone (AddgeneIDs 190114–190119), the Type IIS restriction enzyme BsaI, and T4-DNA ligase (both NEB). In level 1 and 2 plasmids, the cloning site for the TUs disrupts the *mCherry* gene, interfering with its expression such that the successful integration of an ORF would result in the disappearance of *mCherry* and a colony color shift from red to white.

### Cloning of CRISPRi Expression Units

To create a full CRISPRi expression unit, the level 1 plasmids containing *dCas9* and the sgRNAs needed to be combined in a level 2 plasmid. For this, a level 2 Golden Gate reaction using the appropriate plasmids (AddgeneIDs 190120–190125), the Type IIS restriction enzyme BsmBI, T4-DNA ligase, and the previously generated level 1 plasmids were used. Positive clones were screened via red-white colony colors as indicated above. The system allows the expression of up to 20 sgRNAs at the same time.

## Supporting Information Available

Detailed materials and methods for the experiments provided here; example protocols for the design of custom CRISPRi constructs and the cloning of transcriptional reporters; table of oligonucleotides used; table of plasmids used and generated; figures showing stable plasmid replication in *A. baumannii* and transformation efficiency; sequences of pMB04 and pMB05; sequencing results of plasmid used in Supplemental Figure 1 at day 1 and day 5 (PDF)

## Abbreviations

ESKAPE: acronym for six organisms of critical importance in in-hospital infections because of their abilities to gain antibiotic resistances: *Enterococcus faecium*, *Staphylococcus aureus*, *Klebsiella pneumoniae*, *Acinetobacter baumannii*, *Pseudomonas aeruginosa*, and *Enterobacter* spp.
MoClo: modular cloning (plasmid sets specifically created for Golden Gate cloning)
CRISPRi: clustered regularly interspaced short palindromic repeats interference (an expression inhibition system based on a deactivated Cas9 protein physically blocking transcription of a genomic region through an sgRNA binding in that region)
TU: transcriptional unit
egfp: enhanced green fluorescent protein
ATc: anhydrotetracycline
X-Gal: 5-bromo-4-chloro-3-indolyl-β-d-galactopyranoside (an organic compound used for blue-white screening)

## References

[ref1] Blot S.; Vandewoude K.; Colardyn F. Nosocomial bacteremia involving *Acinetobacter baumannii* in critically ill patients: a matched cohort study. Intensive Care Med. 2003, 29, 471–475. 10.1007/s00134-003-1648-8. 12577148

[ref2] Centers for Disease Control and Prevention (U.S.); National Center for Emerging Zoonotic and Infectious Diseases (U.S.). Division of Healthcare Quality Promotion. Antibiotic Resistance Coordination and Strategy Unit. Antibiotic Resistance Threats in the United States, 2019. U.S. Department of Health and Human Services, 2019. https://stacks.cdc.gov/view/cdc/82532. 10.15620/cdc:82532.

[ref3] Ayoub Moubareck C.; Hammoudi Halat D. Insights into *Acinetobacter baumannii*: A Review of Microbiological, Virulence, and Resistance Traits in a Threatening Nosocomial Pathogen. Antibiotics 2020, 9, 119 10.3390/antibiotics9030119. 32178356 PMC7148516

[ref4] Manchanda V.; Sanchaita S.; Singh N. Multidrug Resistant *Acinetobacter*. J. Global Infect. Dis. 2010, 2, 291 10.4103/0974-777X.68538. PMC2946687 20927292

[ref5] Jie J.; Chu X.; Li D.; Luo Z. A set of shuttle plasmids for gene expression in *Acinetobacter baumannii*. PLoS One 2021, 16, e0246918 10.1371/journal.pone.0246918. 33566854 PMC7875395

[ref6] Lucidi M.; Runci F.; Rampioni G.; Frangipani E.; Leoni L.; Visca P. New Shuttle Vectors for Gene Cloning and Expression in Multidrug-Resistant *Acinetobacter* Species. Antimicrob. Agents Chemother. 2018, 62, e02480-17 10.1128/AAC.02480-17. 29339383 PMC5913964

[ref7] Aranda J.; Poza M.; Pardo B. G.; Rumbo S.; Rumbo C.; Parreira J. R.; Rodríguez-Velo P.; Bou G. A rapid and simple method for constructing stable mutants of *Acinetobacter baumannii*. BMC Microbiol. 2010, 10, 279 10.1186/1471-2180-10-279. 21062436 PMC2993698

[ref8] Biswas I. Genetic tools for manipulating *Acinetobacter baumannii* genome: an overview. J. Med. Microbiol 2015, 64, 657–669. 10.1099/jmm.0.000081. 25948809

[ref9] Sykes E. M. E.; Deo S.; Kumar A. Recent Advances in Genetic Tools for *Acinetobacter baumannii*. Front. Genet 2020, 11, 601380 10.3389/fgene.2020.601380. 33414809 PMC7783400

[ref10] Wang Y.; Wang Z.; Chen Y.; Hua X.; Yu Y.; Ji Q. A Highly Efficient CRISPR-Cas9-Based Genome Engineering Platform in *Acinetobacter baumannii* to Understand the H_2_O_2_-Sensing Mechanism of OxyR. Cell Chem. Biol. 2019, 26, 1732–1742. 10.1016/j.chembiol.2019.09.003. 31548010

[ref11] Qi L. S.; Larson M. H.; Gilbert L. A.; Doudna J. A.; Weissman J. S.; Arkin A. P.; Lim W. A. Repurposing CRISPR as an RNA-guided platform for sequence-specific control of gene expression. Cell 2013, 152, 1173–1183. 10.1016/j.cell.2013.02.022. 23452860 PMC3664290

[ref12] Bai J.; Dai Y.; Farinha A.; Tang A. Y.; Syal S.; Vargas-Cuebas G.; van Opijnen T.; Isberg R. R.; Geisinger E. Essential gene analysis in *Acinetobacter baumannii* by high-density transposon mutagenesis and CRISPR interference. J. Bacteriol. 2021, e00565-20 10.1128/JB.00565-20. 33782056 PMC8316057

[ref13] Peters J. M.; Koo B. M.; Patino R.; Heussler G. E.; Hearne C. C.; Qu J.; Inclan Y. F.; Hawkins J. S.; Lu C. H. S.; Silvis M. R.; Harden M. M.; Osadnik H.; Peters J. E.; Engel J. N.; Dutton R. J.; Grossman A. D.; Gross C. A.; Rosenberg O. S. Enabling genetic analysis of diverse bacteria with Mobile-CRISPRi. Nat. Microbiol. 2019, 4, 244–250. 10.1038/s41564-018-0327-z. 30617347 PMC6424567

[ref14] Engler C.; Gruetzner R.; Kandzia R.; Marillonnet S. Golden Gate Shuffling: A One-Pot DNA Shuffling Method Based on Type IIs Restriction Enzymes. PLoS One 2009, 4, e5553 10.1371/journal.pone.0005553. 19436741 PMC2677662

[ref15] Engler C.; Kandzia R.; Marillonnet S. A One Pot, One Step, Precision Cloning Method with High Throughput Capability. PLoS One 2008, 3, e3647 10.1371/journal.pone.0003647. 18985154 PMC2574415

[ref16] Cermak T.; Doyle E. L.; Christian M.; Wang L.; Zhang Y.; Schmidt C.; Baller J. A.; Somia N. V.; Bogdanove A. J.; Voytas D. F. Efficient design and assembly of custom TALEN and other TAL effector-based constructs for DNA targeting. Nucleic Acids Res. 2011, 39, e82 10.1093/nar/gkr218. 21493687 PMC3130291

[ref17] Vad-Nielsen J.; Lin L.; Bolund L.; Nielsen A. L.; Luo Y. Golden Gate Assembly of CRISPR gRNA expression array for simultaneously targeting multiple genes. Cell. Mol. Life Sci. 2016 73:22 2016, 73, 4315–4325. 10.1007/s00018-016-2271-5. PMC11108369 27178736

[ref18] Cress B. F.; Toparlak O. D.; Guleria S.; Lebovich M.; Stieglitz J. T.; Englaender J. A.; Jones J. A.; Linhardt R. J.; Koffas M. A. G. CRISPathBrick: Modular Combinatorial Assembly of Type II-A CRISPR Arrays for dCas9-Mediated Multiplex Transcriptional Repression in *E. coli*. ACS Synth. Biol. 2015, 4, 987–1000. 10.1021/acssynbio.5b00012. 25822415

[ref19] Moore S. J.; Lai H.-E.; Kelwick R. J. R.; Chee S. M.; Bell D. J.; Polizzi K. M.; Freemont P. S. EcoFlex: A Multifunctional MoClo Kit for *E. coli* Synthetic Biology. ACS Synth. Biol. 2016, 5, 1059–1069. 10.1021/acssynbio.6b00031. 27096716

[ref20] Iverson S. v.; Haddock T. L.; Beal J.; Densmore D. M. CIDAR MoClo: Improved MoClo Assembly Standard and New *E. coli* Part Library Enable Rapid Combinatorial Design for Synthetic and Traditional Biology. ACS Synth. Biol. 2016, 5, 99–103. 10.1021/acssynbio.5b00124. 26479688

[ref21] Hunger M.; Schmucker R.; Kishan V.; Hillen W. Analysis and nucleotide sequence of an origin of an origin of DNA replication in *Acinetobacter calcoaceticus* and its use for *Escherichia coli* shuttle plasmids. Gene 1990, 87, 45–51. 10.1016/0378-1119(90)90494-C. 2185139

[ref22] Murin C. D.; Segal K.; Bryksin A.; Matsumura I. Expression Vectors for *Acinetobacter baylyi* ADP1. Appl. Environ. Microbiol. 2012, 78, 280 10.1128/AEM.05597-11. 22020504 PMC3255645

[ref23] Huys G.; Bartie K.; Cnockaert M.; Hoang Oanh D. T.; Phuong N. T.; Somsiri T.; Chinabut S.; Yusoff F. M.; Shariff M.; Giacomini M.; Teale A.; Swings J. Biodiversity of chloramphenicol-resistant mesophilic heterotrophs from Southeast Asian aquaculture environments. Res. Microbiol. 2007, 158, 228–235. 10.1016/j.resmic.2006.12.011. 17350231

[ref24] Lin L.; Ling B. D.; Li X. Z. Distribution of the multidrug efflux pump genes, adeABC, adeDE and adeIJK, and class 1 integron genes in multiple-antimicrobial-resistant clinical isolates of *Acinetobacter baumannii*–*Acinetobacter calcoaceticus* complex. Int. J. Antimicrob. Agents 2009, 33, 27–32. 10.1016/j.ijantimicag.2008.06.027. 18790612

[ref25] Roca I.; Marti S.; Espinal P.; Martínez P.; Gibert I.; Vila J. CraA, a Major Facilitator Superfamily Efflux Pump Associated with Chloramphenicol Resistance in *Acinetobacter baumannii*. Antimicrob. Agents Chemother. 2009, 53, 4013 10.1128/AAC.00584-09. 19581458 PMC2737869

[ref26] Cormack B. P.; Valdivia R. H.; Falkow S. FACS-optimized mutants of the green fluorescent protein (GFP). Gene 1996, 173, 33–38. 10.1016/0378-1119(95)00685-0. 8707053

[ref27] Schneider C. A.; Rasband W. S.; Eliceiri K. W. NIH Image to ImageJ: 25 years of image analysis. Nat. Methods 2012, 9, 671–675. 10.1038/nmeth.2089. 22930834 PMC5554542

[ref28] Schindelin J.; Arganda-Carreras I.; Frise E.; Kaynig V.; Longair M.; Pietzsch T.; Preibisch S.; Rueden C.; Saalfeld S.; Schmid B.; Tinevez J. Y.; White D. J.; Hartenstein V.; Eliceiri K.; Tomancak P.; Cardona A. Fiji: an open-source platform for biological-image analysis. Nat. Methods 2012, 9, 676–682. 10.1038/nmeth.2019. 22743772 PMC3855844

