# Supplementary material for: AspFlex: Molecular Tools to Study Gene Expression and Regulation in Acinetobacter baumannii
Source: ACS Synth Biol. 2023 Aug 16;12(9):2773–7. doi: 10.1021/acssynbio.3c00167 (PMC10621034; doi:10.1021/acssynbio.3c00167)
Supplement: Supplementary file 1 — sb3c00167_si_001.pdf [file sb3c00167_si_001.pdf]

AspFlex: Molecular Tools to Study Gene Expression and Regulation in *Acinetobacter baumannii* supplemental information

Merlin Brychcy[1], Alexis Kokodynski[1], Devin Lloyd[1], Veronica Godoy-Carter[1]

[1]Biology Department, Northeastern University, Boston, MA, 02115, USA

Supplemental information

Bacterial strains and growth conditions

Escherichia coli DH5α was used for all initial molecular cloning. *A. baumannii* ATCC 17978 was prepared as previously reported[1] to introduce plasmids by electroporation. Strains were grown in LB Miller (10 g/L NaCl; 10 g/L Tryptone; 5 g/L yeast extract; and for plates 20 g/L Agar). Selection was performed by addition of necessary antibiotics to plates; for *A. baumannii*: Carbenicillin 100 µg/mL; Kanamycin 15 µg/mL and Tellurite 30 µg/mL. For *E. coli* Chloramphenicol 20 µg/mL; Carbenicillin 100 µg/mL; Kanamycin 15 µg/mL and Tellurite 15 µg/mL. Strains were grown at 37°C at 225 rpm to saturation for 16 h.

Site-directed mutagenesis

For site-directed mutagenesis, the plasmid to be mutagenized was first amplified using Phusion Polymerase (Thermo Fisher Scientific) and primers introducing the point-mutation of interest (to point mutate dCas9 pBP_dCas9_mut_f and pBP_dCas9_mut_r; for the TelR cassette pMo130-TelR_pointBsmBI_f and pMo130_TelR_pointBsmBI_r; Supplemental Table 1). The plasmid amplified by PCR was purified using GeneJet PCR Purification kit (Thermo Fisher Scientific), phosphorylated using T4 PNK (Thermo Fisher Scientific) and circularized using T4 DNA Ligase (NEB). Lastly, the amplified plasmid was digested with DpnI (NEB) to remove the native backbone and introduce into *E. coli* by transformation. Positive clones were confirmed via whole plasmid sequencing (plasmidsaurus.com).

Transformation of *A. baumannii*

Plasmids were introduced into *A. baumannii* via electroporation as previously described[1].

Microscope imaging and fluorescence quantification

To prepare cells for imaging a saturated culture of the appropriate *A. baumannii* strain containing the plasmid of interest was grown for at least 16h at 37°C in LB medium with antibiotic (in this case Kanamycin). The saturated culture was diluted 1:1000 into fresh LB medium (no antibiotic added) with the appropriate concentration of ATc, the inducing agent, until reaching exponential phase (around 3 h). 2 µL of bacterial cells were then deposited on 1% agarose pads on microscope slides[2]. Cells were imaged in a Leica DM5000 DFC3000 G microscope with a 100x magnification. Images were taken in phase contrast, with a Texas red filter (Excitation: 540 nm – 580 nm; Dichromatic mirror: 595 nm; Emission: 607 nm – 683 nm) or a gfp filter (Excitation: 450 nm – 490 nm; Dichromatic mirror: 495 nm; Emission: 500 nm – 550 nm).

To quantify fluorescence in strains with CRISPRi test plasmids pMB04 (targeting sgRNA) and pMB05 (non-targeting sgRNA), single cell fluorescence microscopy was carried out. Images were quantified with ImageJ/Fiji[3]. For this, a custom script was generated (see Supplemental Code 1), which opens both the phase contrast and fluorescence image first and converts both to 16-bit images. To identify cells, the phase contrast image is converted to a Black and White image in which the cells are highlighted using the 'Threshold' function of ImageJ. Connected cells were disconnected using the 'Watershed' function. Fluorescence intensity was measured using the 'Analyze particles' function referencing the fluorescent image for intensity and filtering out small particles. Cells with a relative mean value over 5 were determined to be fluorescent. The fluorescence intensity was measured in 10 sets of microscope images with at least

500 cells per set. The number of fluorescing cells was divided by the total number of cells to obtain the percentage of fluorescent cells[3].

Supplemental Code 1: ImageJ/Fiji script code to analyze fluorescent intensity.

```
//Open the fluorescent image and get filename
path1 = File.openDialog("Select fluorescent file");
  open(path1);
  fluoimage = getInfo("image.filename");
//Convert to 16 bit
  run("16-bit");


//Open phase contrast image (should be 16 bit already)and get filename
  path2 = File.openDialog("Select background file");
  backgroundimage = File.getName(path2);
  open(path2);


//Run threshold to determine what is cell and what is background
setAutoThreshold("Default no-reset");
setOption("BlackBackground", true);
run("Convert to Mask");

//Run watershed to separate connected cells
run("Watershed");

//Redirect phase image to fluorescent image to determine fluo intensity
redirectImageTitle = fluoimage;
//Define what is measured (size of cell and fluorescence)
run("Set Measurements...", "area mean redirect=[" +redirectImageTitle
+"]decimal=3");
//Run actual quantification, set size to >50 to avoid possible small
particles
run("Analyze Particles...", "size=50-Infinity display");

//Save results and close
saveAs("Results");
close("Results");
close("*");
```

Statistical analysis

To test for statistical significance, an ordinary one-way ANOVA analysis with multiple comparisons and a Gaussians distribution was used. Statistical Hypothesis testing was done using Tukey-Test. A confidence

interval of 0.05 was chosen. Values of each concentration were compared to the 0 ng/mL ATc. The statistical analysis was done with GraphPad Prism.

Design and cloning of sgRNAs for CRISPRi and generation of pMB04 and pMB05 plasmids.

The process of generating a complete CRISPRi construct involves multiple steps using AspFlex. The initial stage requires identifying a potential sgRNA site, which is ideally located within the promoter region or close to the 5' end of the open reading frame of the protein target intended for CRISPRi-knockdown. While sgRNAs that are complementary to the non-template strand are preferred, we have also observed effective outcomes with sgRNAs targeting the template strand. Once the appropriate NGG PAM site is identified in the regions mentioned above, the next step involves designing two complementary oligonucleotides specific to that sgRNA. The oligonucleotide situated on the same strand as the NGG site should possess a TAGT 5' overhang, while the complementary oligonucleotide needs an AAAC 5' overhang . For pMB04 these oligonucleotides were: mCherry_sgRNA_f and mCherry_sgRNA_r, and GFP_sgRNA_f and GFP_sgRNA_r respectively (Supplemental Table 1). For pMB05, two oligonucleotides creating a sgRNA not binding in the genome was created leading to oligonucleotides nonsense_sgRNA_f and nonsense_sgRNA_r (Supplemental Table 1).

The two oligonucleotides were annealed to create double stranded DNA with the necessary overhangs for the Golden Gate reaction. For this, 5 µL of 10x T4 DNA ligase buffer (NEB) was combined with 5 µL of each of the oligonucleotides at 100 µM in a PCR reaction tube in a final volume of 50 µL. The reaction tube was incubated in a Thermocycler at 95 °C for 5 minutes, followed by 22 °C for 20 minutes.

A Golden Gate reaction with the annealed oligonucleotides was then set up. The reaction had a final volume of 10 µL and contained 1 µL of T4 DNA ligase buffer (NEB), 1 µL of the annealed oligonucleotides, 0.03 pmol (~ 50 ng) of the pBP_sgRNA plasmid, 0.25 µL of BpiI-HF (NEB) and 0.5 µL of T4 DNA ligase (NEB). The reaction mix was incubated in the thermocycler with the following settings: Cycling 50 times

with a first step at 37 °C (digestion step) for 2 minutes, followed by 16 °C for 5 minutes (ligation step). Afterwards, the sample was incubated for 1 hour at 37 °C to digest left-over backbone plasmid, followed by an 80 °C step for 20 minutes to heat-inactivate the enzymes.

The reaction mixture is then introduced by transformation into *E. coli* competent cells (for example DH5α) and deposited on LB medium plates containing Chloramphenicol, as well as IPTG and X-Gal for blue-white screening. White colonies are confirmed via colony PCR the next day. While the reactions are generally highly efficient and correct, it is still recommended to sequence all plasmids to avoid SNPs. Positive clones yield a successful level 0 sgRNA construct. For pMB04 and pMB05 this generated level 0 plasmids pBP_mcherry_sgRNA, pBP_gfp_sgRNA and pBP_nonsense_sgRNA respectively, each containing the according functional sgRNA unit.

Afterwards, a level 1 plasmid was created with the successfully constructed level 0 sgRNA plasmid. The backbone selected in this case depends on the amount of sgRNAs expressed (see supplemental table 2). Considering that the *dCas9* constructs are present in pMB1-A plasmids, sgRNAs should never be cloned into pMB1-A, because multiple pMB1-A constructs would lead to incompatible overhangs in level 2 plasmids. For a single sgRNA construct, the guide RNA needs to be cloned into pMB1-B. For this in a 10 µL reaction, 30 pmols (~150 ng) of pMB1-B (or accordingly to other level 1 plasmid) with 0.03 pmol (50 ng) of the level 0 construct, 1 µL of T4 DNA ligase buffer, 0.25 µL of BsaI-HFv2 (NEB) and 0.5 µL of T4-DNA ligase (NEB) were combined. Mixes were incubated with the same settings as the level 0 reaction, introduce by transformation into standard *E. coli* competent cells (for example DH5α) and deposited on LB medium plates containing Ampicillin/Carbenicillin as a selection agent. The successful clones are white and were confirmed with Colony-PCR and sequencing. To generate the level 1 sgRNA plasmids, pBP_mcherry_sgRNA was combined with pMB1-B generating pMB1-B_mcherry_sgRNA; pBP_gfp_sgRNA with pMB1-E generating pMB1-E_gfp_sgRNA; and pBP_nonsense_sgRNA was combined with both pMB1-B and pMB1-E generating pMB1-B_nonsense_sgRNA and pMB1-E_nonsense_sgRNA respectively. Additionally, two plasmids were generated with a fluorescent protein

and a strong promoter as indicated next. To generate pMB1-D1_strong_gfp and pMB1-C_strong_mcherry the following plasmids were combined in a level 1 reaction as described above: pBP-ORF_egfp (AddgeneID: 72960), pBP-pET-RBS (AddgeneID: 72981), pBP-Bba_B0012 (AddgeneID: 72997), pBP-SJM910 (AddgeneID: 72972) and pMB1-D1; pBP-ORF_mcherry (AddgeneID: 72962), pBP-pET-RBS, pBP-Bba_B0012. pBP-SJM910 and pMB1-C respectively.

To finish the cloning process, a level 2 plasmid containing both the sgRNAs and the *dCas9* needed to be created. In the case of a single guide RNA, in a 10 μL reaction, 1 μL of T4-DNA ligase buffer is combined with 0.015 pmol (75 ng) of both of the level 1 constructs (in this case the sgRNA in pMB1-B and the *dCas9* in pMB1-A), 0.015 pmol (75 ng) of the level 2 backbone (in this case pMB2a; see supplemental table 2) and 1 μL of NEBridge Golden Gate assembly BsmBI-HFv2 master mix. Mixtures were incubated in the thermocycler with the following settings: 50 cycles at 42 °C for 5 minutes first, then at 16 °C for 5 minutes. Enzymes were heat -inactivated with a cycle at 60 °C for 5 minutes. To generate pMB04 the plasmids included in the reaction were the following: pMB2-A, pMB1-A-ptet-FLAG-dcas9, pMB1-B_mcherry_sgRNA, pMB1-C_strong_mcherry, pMB1-D1_strong_gfp and pMB1-E_gfp_sgRNA. For pMB05 the same plasmids were used, substituting the sgRNA plasmids with the according nonsense sgRNA plasmids.

The reaction mix was then introduced by transformation into competent *E. coli* cells (highly competent commercial cells, for example NEB® 10-beta, are recommended) selecting on LB medium with Kanamycin and screening for white colonies. Colony PCR and sequencing are recommended. A control digest, considering the size of the plasmid, could also be performed. The constructs are introduced by transformation into *A. baumannii* once the sequence is confirmed.

If a level 3 plasmid needs to be created, follow the instructions for a level 1 plasmid.

To induce CRISPRi the optimal concentration of ATc required depends on the level of suppression needed of the protein targeted for knock-down. Generally, a full CRISPRi knockdown can be observed by adding

200 ng/mL of ATc on plates. However, in liquid culture, lower concentrations of ATc (for example 50 ng/mL) can be used. Our recommendation is to first grow a saturated overnight culture with the selective agent (Kanamycin) and then outgrow the cells without Kanamycin with and without ATc until they reach exponential phase. Plasmid stability is insured via a toxin-antitoxin system on the plasmid guaranteeing replication of the plasmid even without the plasmids marker. The successful knock-down can be detected either via qPCR or through phenotypical changes associated with the knock-down.

Example: Cloning of a transcriptional reporter with AspFlex and EcoFlex

To yield the full power of AspFlex, we heavily recommend acquiring the EcoFlex kit by the Freemont lab (Addgene kit #1000000080). This kit will provide additional plasmids expanding the possibilities the AspFlex kit offers, especially regarding expression or reporter plasmids. To create a transcriptional reporter with AspFlex, multiple level 0 elements from EcoFlex can be used. For example, the pET-RBS (AddgeneID: 72981), the *egfp* gene (AddgeneID: 72960) and the Bba_B0012 terminator (AddgeneID: 72997). The promoter region is amplified with primers containing BsaI sites and the according overhangs (CTAT and GTAC). A Golden Gate level 1 reaction like the one described above (use BsaI as restriction and combine all the elements mentioned above with a level 1 plasmid of choice) can be set up and introduced by transformation into *E. coli.* The plasmids can be introduced by transformation into *A. baumannii* to serve as transcriptional reporters after confirmation via colony PCR and sequencing.

Generation of plasmid variants containing tellurite resistance

Additional variants of each plasmid containing an additional tellurite resistance cassette for use in multi-drug resistance *A. baumannii* strains are also available under 'Accessory plasmids' on the Kit's Addgene webpage. We do not recommend using tellurite as a selecting agent during the cloning process, but only when transforming into *A. baumannii*, considering the black color displayed by colonies resistant to tellurite does not allow screen for lack of red fluorescence. Colonies should be stable via the toxin-antitoxin system after initial selection with tellurite.

First, the internal BsmBI site in the tellurite resistance cassette of pMo-130-TelR (AddgeneID: 50799) was mutated via site directed mutagenesis as described above with the primer pair pMo-130-TelR_pointBsmBI_f and pMo-130-TelR_pointBsmBI_r generating pMo-130-TelR-point.

To generate the tellurite resistance variants of the level 1 (pMB1-A-TelR – pMB1-E-TelR) and level 3 plasmids (pMB3-A-TelR and pMB3-B-TelR), the plasmids were digested with XbaI dephosphorylated using QuickCIP (both NEB). Subsequently, pMo-130-TelR-point was digested with XbaI and the fragment consisting of the tellurite resistance cassette was gel-purified. The purified cassette and the according level 1 or level 3 plasmid were then ligated using T4 DNA ligase and transformed into E. coli DH5α. Positive bacterial clones were selected based on their resistance to both tellurite and carbenicillin as well as by observing red fluorescence. Clones were confirmed using control digests with XbaI as well as whole plasmid sequencing.

To generate the level 2 plasmids with a tellurite resistance cassette (pMB2a-TelR – pMB2-D-TelR) the plasmids were digested using NotI-HF and dephosphorylated using QuickCIP (both NEB). The tellurite cassette was amplified with the bi-directional oligonucleotide TelR_NotI_birectional from pMo-130-TelR-point and digested with NotI-Hf afterwards. Ligation and selection of positive clones was done as described above for the level 1 and 3 plasmids.

Testing plasmid stability and replication

To test plasmid stability in A. baumannii, we first generated a plasmid that expresses *gfp* robustly to observe protein expression over time by fluorescence measurements. For this, a level one plasmid containing the SJM910 promoter (AddgeneID: 72972), a pET-RBS (AddgeneID: 72981), an *egfp* (AddgeneID: 72960) and the Bba_B0012 terminator (AddgeneID: 72997) was constructed as described above using a level 1 Golden-Gate reaction and named pMB1-A-strong_gfp. The plasmid was subsequently introduced via transformation into *A. baumannii* ATCC17978 via electroporation. Plasmid stability was determined in two

different ways: First, a culture containing the plasmid was outgrown to post-logarithmic phase and subsequently diluted 1:1000 every 24h. At every 24h time point, the culture's fluorescence intensity was observed via microscope imaging and further image analysis as described above. To show stable replication through the toxin-antitoxin present in the plasmids, we grew the culture both with and without the according selecting agent (Carbenicillin).

Additionally, to show that the plasmids did not go through any type of recombination, all plasmids were extracted from *A. baumannii* using ThermoFisher GeneJet Plasmid Miniprep kit at day 0 and 5 and subsequently fully sequenced. Plasmids containing the cloned construct were aligned to the plasmids original sequence using MUSCLE[4].

Determination of cloning efficiency

To determine cloning efficiency, an example level 0, level 1, and level 2 cloning of a CRISPRi unit was used. For this, the two nonsense sgRNA oligonucleotides were first cloned into level 0 plasmids as described earlier. After, they were cloned into level 1 and combined with the level 1 dCas9 plasmid into a level 2 plasmid, both as following instructions above. Each cloning was repeated 3 times. For the determination of the efficiency, colonies with their according phenotype (color) were counted. Additionally, plasmids from 8 colonies in each level exhibiting white color were extracted and subsequently sequenced.

Oligonucleotides

Supplemental Table 1: Oligonucleotides used in this work.

Oligonucleotide name	Sequence (5' - 3')
ori_ab_pstI_r	AAATTCTGCAGTTAACAAGTTGCCTGACGCC
ori_ab_pstI_f	ATATTCTGCAGTTGCAAGACAATATCGACCG
pCas9_new_r	attggtctcttcgagttagtcacctcctagctgac
pCas9_new_f	attGGTCTCacataatggataagaaatactcaataggcttag
pBP_cas9_mut_r	ttccgctgtctctccactgt
pBP_cas9_mut_f	gcgactcgtcttaaacggac
mCherry_sgRNA_f	tagtcaagggcgaggaggataaca
mCherry_sgRNA_r	aaactgttatcctcctcgcccttg
GFP_sgRNA_r	aaacgtgaaaagttcttctccttt
GFP_sgRNA_f	tagtaaaggagaagaacttttcac
nonsense_sgRNA_r	aaacCTGCCATACCAGGCGCGTAC
nonsense_sgRNA_f	tagtGTACGCGCCTGGTATGGCAG
seq_primer	Atttcagataaaaaaaatccttagctttcg
pMo130-TelR_pointBsmBI_f	tcgacggacgtgcgat
pMo130-TelR_pointBsmBI_r	Aacggcgaaggagaaccaac
TelR_NotI_birectional	atatGCGGCCGCcccgggtaccgag

Plasmids

Supplemental Table 2: Plasmids used in this work. *mcherry*, red fluorescent protein; *lacZ*, alpha peptide.

Plasmid name	Level	Purpose	Antibiotic marker	Screening marker	AddgeneID	Restriction enzyme	Source
pMB1-A	1	Cloning of single TU	Carbenicillin	*mcherry*	190114	BsaI	This study
pMB1-B	1	Cloning of single TU	Carbenicillin	*mcherry*	190115	BsaI	This study
pMB1-C	1	Cloning of single TU	Carbenicillin	*mcherry*	190116	BsaI	This study
pMB1-D	1	Cloning of single TU	Carbenicillin	*mcherry*	190117	BsaI	This study
pMB1-D1	1	Cloning of single TU	Carbenicillin	*mcherry*	190118	BsaI	This study
pMB1-E	1	Cloning of single TU	Carbenicillin	*mcherry*	190119	BsaI	This study
pMB2a	2	Cloning of 2 TUs	Kanamycin	*mcherry*	190120	BsmBI	This study
pMB2b	2	Cloning of 3 TUs	Kanamycin	*mcherry*	190121	BsmBI	This study
pMB2-A	2	Cloning of 4 or 5 TUs	Kanamycin	*mcherry*	190122	BsmBI	This study
pMB2-B	2	Cloning of 4 or 5 TUs	Kanamycin	*mcherry*	190123	BsmBI	This study
pMB2-C	2	Cloning of 4 or 5 TUs	Kanamycin	*mcherry*	190124	BsmBI	This study
pMB2-D	2	Cloning of 4 or 5 TUs	Kanamycin	*mcherry*	190125	BsmBI	This study
pMB3-A	3	Cloning of 2 fragments from level 2	Carbenicillin	*mcherry*	190126	BsaI	This study
pMB3-B	3	Cloning of 4 fragments from level 2	Carbenicillin	*mcherry*	190127	BsaI	This study
pBP_sgRNA	0	Cloning of level 0 sgRNA	Chloramphenicol	*lacZ*	190128	BpiI	This study
pMB1-A-ptet-dCas9	1	Level 1 plasmid expressing ATc inducible dCas9	Carbenicillin		190129	BsmBI	This study
pMB1-A-ptet-FLAG-dCas9	1	Level 1 plasmid expressing ATc inducible FLAG tagged dCas9	Carbenicillin		190130	BsmBI	This study
pBP_dCas9	0	Level 0 plasmid containing point mutated dCas9	Chloramphenicol		190131	BsaI	This study
pMB04	2	Proof of principle sgRNAs targeting the fluorescence proteins	Kanamycin				This study
pMB05	2	Proof of priniciple, nonsense sgRNAs	Kanamycin				This study
pMB1-A-TelR	1	Cloning of single TU, use in multi-drug res. *A. baumannii*	Carbenicillin, Tellurite	*mcherry*	204979	BsaI	This study
pMB1-B-TelR	1	Cloning of single TU, use in multi-drug res. *A. baumannii*	Carbenicillin, Tellurite	*mcherry*	204980	BsaI	This study
pMB1-C-TelR	1	Cloning of single TU, use in multi-drug res. *A. baumannii*	Carbenicillin, Tellurite	*mcherry*	204981	BsaI	This study
pMB1-D-TelR	1	Cloning of single TU, use in multi-drug res. *A. baumannii*	Carbenicillin, Tellurite	*mcherry*	204982	BsaI	This study
pMB1-D1-TelR	1	Cloning of single TU, use in multi-drug res. *A. baumannii*	Carbenicillin, Tellurite	*mcherry*	204983	BsaI	This study
pMB1-E-TelR	1	Cloning of single TU, use in multi-drug res. *A. baumannii*	Carbenicillin, Tellurite	*mcherry*	204986	BsaI	This study

pMB2a-TelR	2	Cloning of 2 TUs, use in multi-drug res. *A. baumannii*	Kanamycin, Tellurite	*mcherry*	204985	BsmBI	This study
pMB2b-TelR	2	Cloning of 3 TUs, use in multi-drug res. *A. baumannii*	Kanamycin, Tellurite	*mcherry*	204986	BsmBI	This study
pMB2-A-TelR	2	Cloning of 4 or 5 TUs, use in multi-drug res. *A. baumannii*	Kanamycin, Tellurite	*mcherry*	204987	BsmBI	This study
pMB2-B-TelR	2	Cloning of 4 or 5 TUs, use in multi-drug res. *A. baumannii*	Kanamycin, Tellurite	*mcherry*	204988	BsmBI	This study
pMB2-C-TelR	2	Cloning of 4 or 5 TUs, use in multi-drug res. *A. baumannii*	Kanamycin, Tellurite	*mcherry*	204989	BsmBI	This study
pMB2-D-TelR	2	Cloning of 4 or 5 TUs, use in multi-drug res. *A. baumannii*	Kanamycin, Tellurite	*mcherry*	204990	BsmBI	This study
pMB3-A-TelR	3	Cloning of 2 fragments from level 2, use in multi-drug res. *A. baumannii*	Carbenicillin, Tellurite	*mcherry*	204991	BsaI	This study
pMB3-B-TelR	3	Cloning of 4 fragments from level 2, use in multi-drug res. *A. baumannii*	Carbenicillin, Tellurite	*mcherry*	204992	BsaI	This study
pWH1266		Source of replication origin for Acinetobacter species					Hunger et al., 1990
pMB1-A-strong_gfp	1	Test plasmid for plasmid stability	Carbenicillin				This study
EcoFlex MoClo kit		Base of plasmids			1000000080		Moore et al., 2016
pdCas9-bacteria		Base plasmid for dCas9 gene	Chloramphenicol		44249		Qi et al., 2013
pMo-130-TelR		Base plasmid for tellurite resistance cassette	Kanamycin, Tellurite		50799		Amin et al., 2013
pMo-130-TelR-point		Point mutated pMo-130-TelR without BsmBI site	Kanamycin, Tellurite				This study
pBP_mcherry_sgRNA	0	Level 0 plasmid containing *mcherry* sgRNA	Chloramphenicol				This study
pBP_gfp_sgRNA	0	Level 0 plasmid containing *gfp* sgRNA	Chloramphenicol				This study
pMB1-B_mcherry_sgRNA	1	Level 1 plasmid containing *mcherry* sgRNA	Carbenicillin				This study
pMB1-E_gfp_sgRNA	1	Level 1 plasmid containing *gfp* sgRNA	Carbenicillin				This study
pMB1-C_strong_mcherry	1	Level 1 plasmid strongly expressing *mcherry*	Carbenicillin				This study
pMB1-D1_strong_gfp	1	Level 1 plasmid strongly expressing *gfp*	Carbenicillin				This study
pBP_nonsense_sgRNA	0	Level 0 plasmid containing nonsense sgRNA	Chloramphenicol				This study
pMB1-B_nonsense_sgRNA	1	Level 1 plasmid containing nonsense sgRNA	Carbenicillin				This study
pMB1-E_nonsense_sgRNA	1	Level 1 plasmid containing nonsense sgRNA	Carbenicillin				This study

Supplemental Table 3: Table showing which AspFlex plasmids are needed to create new plasmids with more than one transcriptional unit (TUs).

Amount of TUs	Plasmids needed	Alternative plasmids (same level)
1	pMB1-A	pMB1-B; pMB1-C; pMB1-D; pMB1-D1; pMB1-E
2	pMB1-A, pMB1-B, pMB2-a	
3	pMB1-A, pMB1-B, pMB1-C, pMB2-b	
4	pMB1-A, pMB1-B, pMB1-C, pMB1-D, pMB2-A	pMB2-B; pMB2-C; pMB2-D
5	pMB1-A, pMB1-B, pMB1-C, pMB1-D1, pMB1-E, pMB2-A	pMB2-B; pMB2-C; pMB2-D
6	pMB1-A, pMB1-B, pMB2-a, pMB1-A, pMB1-B, pMB1-C, pMB1-D, pMB-2B, pMB3-A	
7	pMB1-A, pMB1-B, pMB2-a, pMB1-A, pMB1-B, pMB1-C, pMB1-D1, pMB1-E, pMB2-B, pMB3-A	
8	pMB1-A, pMB1-B, pMB1-C, pMB1-D, pMB2-A, pMB1-A, pMB1-B, pMB1-C, pMB1-D, pMB2-B, pMB3-A	
9	pMB1-A, pMB1-B, pMB1-C, pMB1-D1, pMB1-E, pMB2-A, pMB1-A, pMB1-B, pMB1-C, pMB1-D, pMB2-B, pMB3-A	
10	pMB1-A, pMB1-B, pMB1-C, pMB1-D1, pMB1-E, pMB2-A, pMB1-A, pMB1-B, pMB1-C, pMB1-D1, pMB1-E, pMB2-B, pMB3-A	
	See EcoFlex Kit manual for further guidance with more fragments.	

Supplemental Figures

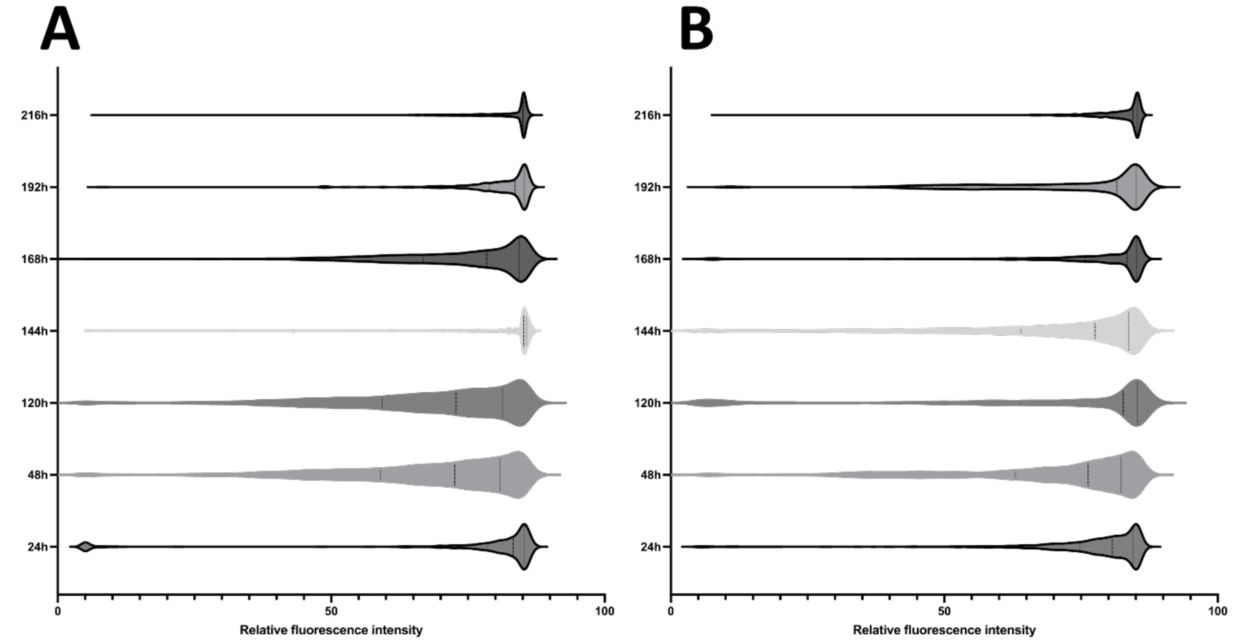

Supplemental Figure 1: AspFlex plasmids show stable replication in *A. baumannii* after multiple generations. The plasmid pMB1-A (AddgeneID: 190114) containing a strongly expressed green fluorescent protein, *egfp*, was introduced by transformation into *A. baumannii* ATCC17978. Plasmid stability was determined by diluting the culture 1:1000 dilution every 24h for 216h in total and measuring the number of fluorescent cells by microscopy quantification using Image-J/Fiji[3]. (A) Cells were passed through multiple subcultures with no selection agent. Most cells continue to be fluorescent suggesting the successful continuous replication of the plasmid. A loss of fluorescent would have led to an accumulation of low/no fluorescent cells. Replication without antibiotic selection is facilitated through a plasmid maintenance toxin-antitoxin system present on the plasmid. (B) A control culture of the same cells in which multiple passage was performed as in A, but with Carbenicillin added as a selective agent (100 µg/mL) for plasmid maintenance. The similarity among the survivors shown in A and B suggests that the plasmid pMB1-A is stably maintained and expresses *gfp* in *A. baumannii*.

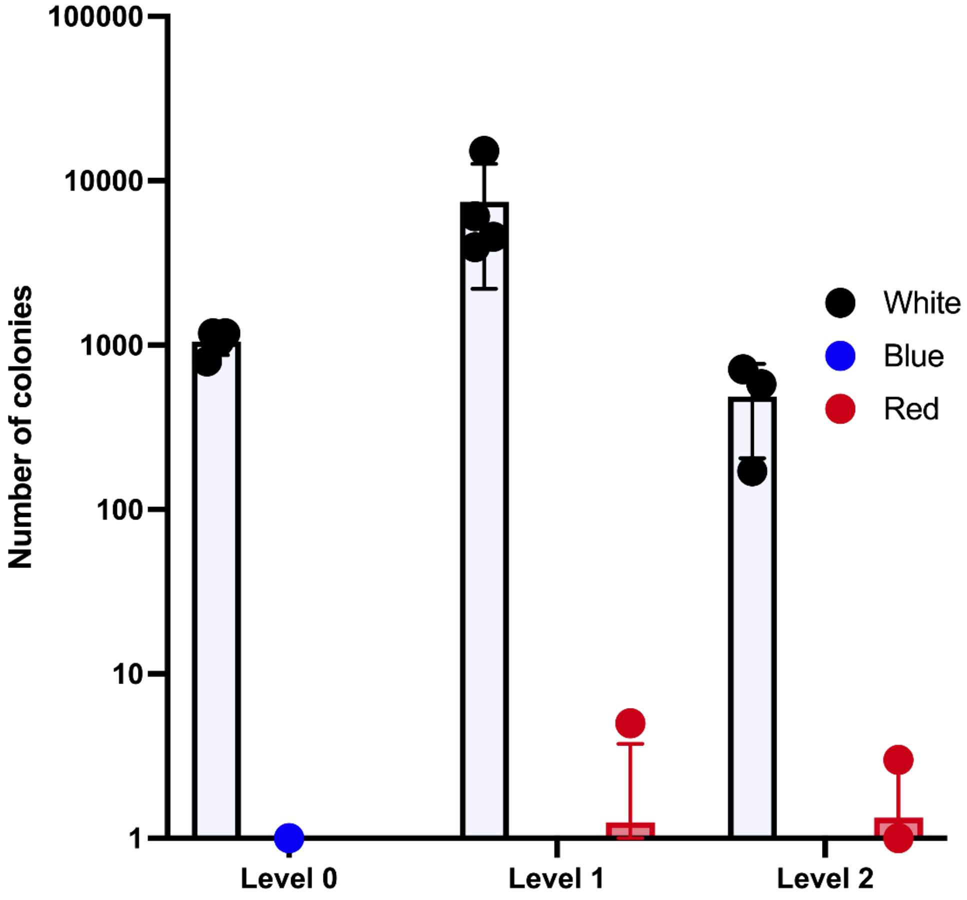

Supplemental Figure 2: Cloning into AspFlex plasmids is highly efficient. The cloning efficiency into level 0-2 was determined by introducing the Golden Gate reaction mixture into *E. coli* DH5α. The mixes were selected on LB-agar plates containing the appropriate antibiotic for each level (Chloramphenicol for level 0; Carbenicillin for level 1; kanamycin for level 2). No-insert colonies are expected to show an antibiotic resistant blue phenotype in level 0 and a red phenotype in level 1 and 2. Level 0 showed no blue phenotype colonies. Eight white colonies were sequenced, confirming the correct insertion of the desired gene. Moreover, no incorrect assembled insert was found in any of the white colonies tested.

Supplemental Sequences

Legend:

Acinetobacter ori; TetR-TetRpromoter-*dCas9*; gfp with promoter and terminator; mcherry with promoter and terminator; mcherry-sgRNA; gfp-sgRNA; Kanamycin resistance cassette; Nonsense-sgRNA

>pMB04 (14,127bp)

ggtctcactatatctctatttaagacccactttcacatttaagttgtttttctaatccgcatatgatcaa
ttcaaggccgaataagaaggctggctctgcaccttggtgatcaaataattcgatagcttgtcgtaataat
ggcggcatactatcagtagtaggtgtttccctttcttctttagcgacttgatgctcttgatcttccaata

cgcaacctaaagtaaaatgccccacagcgctgagtgcatataatgcattctctagtgaaaaaccttgttg
gcataaaaaggctaattgattttcgagagtttcatactgtttttctgtaggccgtgtacctaaatgtact
tttgctccatcgcgatgacttagtaaagcacatctaaaacttttagcgttattacgtaaaaaatcttgcc
agctttccccttctaaagggcaaaagtgagtatggtgcctatctaacatctcaatggctaaggcgtcgag
caaagcccgcttattttttacatgccaatacaatgtaggctgctctacacctagcttctgggcgagttta
cgggttgttaaaccttcgattccgacctcattaagcagctctaatgcgctgttaatcactttacttttat
ctaatctagacatcattaattcctaatttttgttgacactctatcgttgatagagttattttaccactcc
ctatcagtgatagagaaaagaattcaaaagatctgtactttaactttaagaaggagatatataaaATGGA
CTACAAAGACCATGACGGTGATTATAAAGATCATGACATCGATTACAAGGATGACGATGACAAGCTCcat
atggataagaaatactcaataggcttagctatcggcacaaatagcgtcggatgggcggtgatcactgatg
aatataaggttccgtctaaaaagttcaaggttctgggaaatacagaccgccacagtatcaaaaaaaatct
tataggggctcttttatttgacagtggagagacagcggaagcgactcgtcttaaacggacagctcgtaga
aggtatacacgtcggaagaatcgtatttgttatctacaggagatttttcaaatgagatggcgaaagtag
atgatagtttctttcatcgacttgaagagtcttttttggtggaagaagacaagaagcatgaacgtcatcc
tatttttggaaatatagtagatgaagttgcttatcatgagaaatatccaactatctatcatctgcgaaaa
aaattggtagattctactgataaagcggatttgcgcttaatctatttggccttagcgcatatgattaagt
ttcgtggtcatttttttgattgagggagatttaaatcctgataatagtgatgtggacaaactatttatcca
gttggtacaaacctacaatcaattatttgaagaaaaccctattaacgcaagtggagtagatgctaaagcg
attctttctgcacgattgagtaaatcaagacgattagaaaatctcattgctcagctccccggtgagaaga
aaaatggcttatttgggaatctcattgctttgtcattgggtttgacccctaattttaaatcaaattttga
tttggcagaagatgctaaattacagctttcaaaagatacttacgatgatgatttagataatttattggcg
caaattggagatcaatatgctgatttgtttttggcagctaagaatttatcagatgctattttactttcag
atatcctaagagtaaatactgaaataactaaggctcccctatcagcttcaatgattaaacgctacgatga
acatcatcaagacttgactcttttaaaagctttagttcgacaacaacttccagaaaagtataaagaaatc
ttttttgatcaatcaaaaaacggatatgcaggttatattgatgggggagctagccaagaagaattttata
aatttatcaaaccaattttagaaaaaatggatggtactgaggaattattggtgaaactaaatcgtgaaga
tttgctgcgcaagcaacggacctttgacaacggctctattccccatcaaattcacttgggtgagctgcat
gctattttgagaagacaagaagacttttatccatttttaaaagacaatcgtgagaagattgaaaaaatct
tgacttttcgaattccttattatgttggtccattggcgcgtggcaatagtcgttttgcatggatgactcg
gaagtctgaagaaacaattaccccatggaattttgaagaagttgtcgataaaggtgcttcagctcaatca
tttattgaacgcatgacaaactttgataaaaatcttccaaatgaaaaagtactaccaaaacatagtttgc
tttatgagtattttacggtttataacgaattgacaaaggtcaaatatgttactgaaggaatgcgaaaacc
agcatttctttcaggtgaacagaagaaagccattgttgatttactcttcaaaacaaatcgaaaagtaacc
gttaagcaattaaaagaagattatttcaaaaaaatagaatgttttgatagtgttgaaatttcaggagttg
aagatagatttaatgcttcattaggtacctaccatgatttgctaaaaattattaaagataaagatttttt
ggataatgaagaaaatgaagatatcttagaggatattgttttaacattgaccttatttgaagatagggag
atgattgaggaaagacttaaaacatatgctcacctctttgatgataaggtgatgaaacagcttaaacgtc
gccgttatactggttggggacgtttgtctcgaaaattgattaatggtattagggataagcaatctggcaa
aacaatattagatttttttgaaatcagatggttttgccaatcgcaattttatgcagctgatccatgatgat
agtttgacatttaaagaagacattcaaaaagcacaagtgtctggacaaggcgatagtttacatgaacata
ttgcaaatttagctggtagccctgctattaaaaaaggtattttacagactgtaaaagttgttgatgaatt
ggtcaaagtaatggggcggcataagccagaaaatatcgttattgaaatggcacgtgaaaatcagacaact
caaaagggccagaaaaattcgcgagagcgtatgaaacgaatcgaagaaggtatcaaagaattaggaagtc
agattcttaaagagcatcctgttgaaaatactcaattgcaaaatgaaaagctctatctctattatctcca
aaatggaagagacatgtatgtggaccaagaattagatattaatcgtttaagtgattatgatgtcgatgcc
attgttccacaaagtttccttaaagacgattcaatagacaataaggtcttaacgcgttctgataaaaatc
gtggtaaatcggataacgttccaagtgaagaagtagtcaaaaagatgaaaaactattggagacaacttct
aaacgccaagttaatcactcaacgtaagtttgataatttaacgaaagctgaacgtggaggtttgagtgaa

cttgataaagctggttttatcaaacgccaattggttgaaactcgccaaatcactaagcatgtggcacaaa
ttttggatagtcgcatgaatactaaatacgatgaaaatgataaacttattcgagaggttaaagtgattac
cttaaaatctaaattagtttctgacttccgaaaagatttccaattctataaagtacgtgagattaacaat
taccatcatgcccatgatgcgtatctaaatgccgtcgttggaactgctttgattaagaaatatccaaaac
ttgaatcggagtttgtctatggtgattataaagtttatgatgttcgtaaaatgattgctaagtctgagca
agaaataggcaaagcaaccgcaaaatatttcttttactctaatatcatgaacttcttcaaaacagaaatt
acacttgcaaatggagagattcgcaaacgccctctaatcgaaactaatggggaaactggagaaattgtct
gggataaagggcgagattttgccacagtgcgcaaagtattgtccatgccccaagtcaatattgtcaagaa
aacagaagtacagacaggcggattctccaaggagtcaattttaccaaaaagaaattcggacaagcttatt
gctcgtaaaaaagactgggatccaaaaaaatatggtggttttgatagtccaacggtagcttattcagtcc
tagtggttgctaaggtggaaaaagggaaatcgaagaagttaaaatccgttaaagagttactagggatcac
aattatggaaagaagttcctttgaaaaaaatccgattgactttttagaagctaaaggatataaggaagtt
aaaaaagacttaatcattaaactacctaaatatagtctttttgagttagaaaacggtcgtaaacggatgc
tggctagtgccggagaattacaaaaaggaaatgagctggctctgccaagcaaatatgtgaatttttttata
tttagctagtcattatgaaaagttgaagggtagtccagaagataacgaacaaaaacaattgtttgtggag
cagcataagcattatttagatgagattattgagcaaatcagtgaattttctaagcgtgttattttagcag
atgccaatttagataaagttcttagtgcatataacaaacatagagacaaaccaatacgtgaacaagcaga
aaatattattcatttatttacgttgacgaatcttggagctcccgctgcttttaaatattttgatacaaca
attgatcgtaaacgatatacgtctacaaaagaagttttagatgccactcttatccatcaatccatcactg
gtctttatgaaacacgcattgatttgagtcagctaggaggtgactaactcgatcacactggctcaccttc
gggtgggcctttctgcgtttatatgtttgccctatcGATCTTTGACAGCTAGCTCAGTCCTAGGTATAAT
ACtagtcaagggcgaggaggataacaGTTTTAGAGCTAGAAATAGCAAGTTAAAATAAGGCTAGTCCGTT
ATCAACTTGAAAAAGTGGCACCGAGTCGGTGCTTTTTTTGAAGCTTtgttccggctatttgatggctagc
tcagtccttggtattatgctagcgtactttaactttaagaaggagatatacatatggtgagcaagggcga
ggaggataacatggccatcatcaaggagttcatgcgcttcaaggtgcacatggagggctccgtgaacggc
cacgagttcgagatcgagggcgagggcgagggccgcccctacgagggcacccagaccgccaagctgaagg
tgaccaagggtggccccctgcccttcgcctgggacatcctgtcccctcagttcatgtacggctccaaggc
ctacgtgaagcaccccgccgacatccccgactacttgaagctgtccttccccgagggcttcaagtgggag
cgcgtgatgaacttcgaggacggcggcgtggtgaccgtgacccaggactcctccttgcaggacggcgagt
tcatctacaaggtgaagctgcgcggcaccaacttcccctccgacggccccgtaatgcagaagaagaccat
gggctgggaggcctcctccgagcggatgtaccccgaggacggcgccctgaagggcgagatcaagcagagg
ctgaagctgaaggacggcggccactacgacgctgaggtcaagaccacctacaaggccaagaagcccgtgc
agctgcccggcgcctacaacgtcaacatcaagttggacatcacctcccacaacgaggactacaccatcgt
ggaacagtacgaacgcgccgagggccgccactccaccggcggcatggacgagctgtacaagtaaggatcc
tcgatcacactggctcaccttcgggtgggcctttctgcgtttatatgttgaagctatttgatggctagct
cagtccttggtattatgctagcgtactttaactttaagaaggagatatacatatgcgtaaaggagaagaa
cttttcactggagttgtcccaattcttgttgaattagatggtgatgttaatgggcacaaattttctgtca
gtggagagggtgaaggtgatgcaacatacggaaaacttacccttaaatttatttgcactactggaaaact
acctgttccatggccaacacttgtcactactttcggttatggtgttcaatgctttgcgagatacccagat
catatgaaacagcatgactttttcaagagtgccatgcccgaaggttatgtacaggaaagaactatatttt
tcaaagatgacgggaactacaagacacgtgctgaagtcaagtttgaaggtgatacccttgttaatagaat
cgagttaaaaggtattgattttaaagaagatggaaacattcttggacacaaattggaatacaactataac
tcacacaatgtatacatcatggcagacaaacaaaagaatggaatcaaagttaacttcaaaattagacaca
acattgaagatggaagcgttcaactagcagaccattatcaacaaaatactccaattggcgatggccctgt
ccttttaccagacaaccattacctgtccacacaatctgccctttcgaaagatcccaacgaaaagagagat
cacatggtccttcttgagtttgtaacagctgctgggattacacatggcatggatgaactatacaaataat
cgatcacactggctcaccttcgggtgggcctttctgcgtttatatgttcttcctatcGATCTTTGACAGC
TAGCTCAGTCCTAGGTATAATACtagtaaaggagaagaacttttcacGTTTTAGAGCTAGAAATAGCAAG

TTAAAATAAGGCTAGTCCGTTATCAACTTGAAAAAGTGGCACCGAGTCGGTGCTTTTTTTGAAGCTTtgt
tTTAGgtacagagaccctgcaGTTGCAAGACAATATCGACCGcttgcagcttgataaagtgatcaaagag
ggacgagcagctacagcactgctaagggagatcggagagccactgccgccaccagtgaaagtgaaaacag
gttggtttagtgctgattattacggctttgaggacatgctcacacgttgggataatgatcaatctagcct
ttccatttatcacagagagataaaagaggagcaacaggaacagcaacgaaaacaacagcagatagaaaga
gaaactaaacaaaaacaggaagatgacaaaaagaaacggcaggactatgaaagatatttatcaaaatacg
gctatgtcaaagatgctgagtatgaccatatcagcgaccatgttgcttcagacatttccatgtttacacg
tcttcaggcgcaggcatgggcaagtaacgatatgcaagagtacataaggtgttcaaagcaaaaatacgag
cagatcagcgacagaatagcctatgagcgtgacttgaaaaaaatcgggcaattgagccgtatcctggata
aagacagacaggcacttgctgatattttaccgcagttgatgccttactatgagcagatgcaacaggctgt
taataaacggaaaatttacttgagaatgagcgacagccatcatttaaaccacgttatgaccaagaacag
cctaagccgaaaaaagacaatgacctaactttttaatgtgttacaatagcattcaaatgtaacacattgg
agtgaaaaacatgacagaagcgacttttacctttcgggtagaccatgacctaaagcaagaattttcaagc
cttgctaagactgttgatcggtcgggggcgcagctaatccgtgacttcatgcgtgactttgttaagaagc
aacaggaagccgcagactatgacaaatggttcaagcagcaggtacagatcggcttaaatgaagccaatgc
aggcaagttgattccacatgaggacgtaaaggcagagtttgccgctagacgtgctgcaacattggcgaaa
ctggcagcgaagaatgaagattgagtggactgaaacagctcgccaagatcgtagaaatatctatgattat
cttgaagaacgcaaccctatagcagctattgaaattgatgatttaattgaagaaaagacagatttacttg
ttgataatcgactgatgggcgcacaggcagacagaaagatactagggagttagtgatacatccgcatta
tgtggttgtatatgacatcactgatataatacggatactcagagtgctacacacatcgcaggagtggtca
tgacttactcatgtactttggattatttagtgttataaaatcctgatttataaatttttttttgttaaaaa
agataaaagccccttgcaattgcttggggctttaccgtaatttatggggtacagatcttcgatactgaca
tatcggcaatcgaaagcattaaggtttgacgaccgctaatgatttcaccacaggggcttaatgtacctgt
cttaaattctaaggttttaactcgctttgtcaagcatagaccccaaaaatttagccaatgtctgtaactc
aatctgtccatgtgtgggtgatgaggtacagtgacgctagcacacatcggaaaaacgctattactagggg
aactgaacagagtagcggacgcaatgagtagtcatttaattggcggttatgagcgtgttcaggcggtgct
atcaatcgtaatcataacagtggcagcttgatacagtgatgtcatccctgatgcgaaagcgaccgaccga
cggtacatcgaatgggaatactttagggtgatttttaagaatcgctctagggtgagtatttcccattcag
ctctgctccctccctctggtactttaatcaaaagcactactaaacatatgtttttaaataaaaaatattg
atatagagataatattagtaagaataattaaacaattgaatatagataaatcattgttaaataaagatta
attattaaaatgaatgtatacttatatataaatcaatgatttaaaatatttgataaagaaaacttttcaa
aaaaaatataattgagattgtgtcatttcggtcaattcttaatatgttccacgcaagttttagctatggt
gctaaacagaaatttgctgaaaaagaacttttcactgaactggttaaaatgtaagcagcctgagagccgc
caaaaattttaaaaacaaaccgccttaatcatcttcaaaaaatacctctaaaacctcaccatttgcgttt
taagacccatatttcatcctgcccttatgttcccatgctgatagctataaagtgtctgtaatcgcttcct
atgacgttctaggctgttgataacttttggaacaacgcaaaatgttaaaatccgatcatttttttaaccta
gttattttcgttacagggtaactttggtagtattatttcaatatttagttgttacaggataacaaaatat
atgttacagggtaattaatttagcgttacaggataactatagttacagggtaacgttaaatagttatcct
gtaatttaaaaatagttgcaggatagcataaattttacaaataaattttataaaaagttatcctgtaac
taaaaacaatgttatcctgtaactatgatatactatatgggagttaaagacatgaaagacccgaacgatc
agaaaacacaggacatgctaaaagagccgccaaaaaccaatgccgaacgacaaaaagcatatcgtgaaaa
acgcaaaagccttgatagtcaacgtttggaggtgtttatagataagggtgtgtcggatatgcttgcggac
atggtgggcgcagcaggggagagccaaaaagccattttgaccgcattgattgagaaagagtataagcggc
tgtatgcagtgaaaaaatagcaagctaaaaagtacattgattcaaaaagtaatgcagataaaaaagaaa
ccctcgatttatgaggttttttataactactaatgcctatgaaaataagtgcttatcattttctctaat
gtacccaataaaatcggggttttgttccacaagtttcttgatgtcattatcatgtttcttcagtaagtaa
gccccgatcaggatcttgcgttttgcatcgtctgaccttgcctgtttttgctctcgacctttcaccacag
cgtcagctagatcctttcttgccttttcctgacgttccaatttttccttttcttcatcgagcatttgacc

caattccatagctgtttttttgaccatttttaatgcctgtgtctcaacctattttccgaagtatgaacca
tcatcaatgtgaaaagtgttctacttcaaaaactcgacttacatcaagatccgagcgcagcgagcaaaat
caaaaaacaaagtcaaagccttattgctcttgcttttgcttttagcctcgcagagttcccgaaggcgca
cttacgcaaaattttgctacgccaaatttgcaagtacggtcagggaaaccccgacaccccaaccgccc
aaaacttggggcggctgttaataaacaggtgatgaaaatggcaatttaccattgtgaaatgcagaacatt
tcgaggtcagatggtcgctcaatcgtggcatgtgcagcataccgagcaggcgaaaaattgtactgtgata
cgtacggaaaagagcaggactacaccaaaaaaacaggcattgaatacacccaattttgccccaactgg
ggcaagtcctgacatgttagatcgtcaaaccctatggaatcgggtagagcaatccgaactaaaaagaac
ggtgacatcaaacaggaggcaagattagcaaaggaagtagagatagcattgccgcatgaactggataaga
cacagcgtcaggcacttgttaccgagttgtgccagtccttagttaaagcctatggagtggcggtggacgt
agcgatccatgcccctcatgtgcatggggaaggagaaagaaacctcacgcccacataagacacaGGCGT
CAGGCAACTTGTTAACTGCAgtccggcaaaaaagggcaaggtgtcaccaccctgccctttttctttaaaa
ccgaaaagattacttcgcgttatgcaggcttcctcgctcactgactcgctgcgctcggtcgttcggctgc
ggcgagcggtatcagctcactcaaaggcggtaatacggttatccacagaatcaggggataacgcaggaaa
gaacatgtgagcaaaaggccagcaaaaggccaggaaccgtaaaaaggccgcgttgctggcgtttttccac
aggctccgcccccctgacgagcatcacaaaaatcgacgctcaagtcagaggtggcgaaacccgacaggac
tataaagataccaggcgtttccccctggaagctccctcgtgcgctctcctgttccgaccctgccgcttac
cggatacctgtccgcctttctcccttcgggaagcgtggcgctttctcatagctcacgctgtaggtatctc
agttcggtgtaggtcgttcgctccaagctgggctgtgtgcacgaaccccccgttcagcccgaccgctgcg
ccttatccggtaactatcgtcttgagtccaacccggtaagacacgacttatcgccactggcagcagccac
tggtaacaggattagcagagcgaggtatgtaggcggtgctacagagttcttgaagtggtggcctaactac
ggctacactagaagaacagtatttggtatctgcgctctgctgaagccagttaccttcggaaaaagagttg
gtagctcttgatccggcaaacaaaccaccgctggtagcggtggtttttttgtttgcaagcagcagattac
gcgcagaaaaaaaggatctcaagaagatcctttgatcttttctacggggtctgacgctcagtggaacgaa
aactcacgttaagggattttggtcatgagattatcaaaaaggatcttcacctagatccttttaaattaaa
aatgaagttttaaatcaatctaaagtatatatgagtaaacttggtctgacagctcgaggcttggattctc
accaataaaaaacgcccggcggcaaccgagcgttctgaacaaatccagatggagttctgaggtcattact
ggatctatcaacaggagtccaagcgagctcgatattCTCGAGAAGCTGGGGATCCGTTTGATTTTTAATG
GATAATGTGATATAATCTTTAAATACTGTAGAAAAGAGGAAGGAAATAATAAATGGCTAAAATGAGAATA
TCACCGGAATTGAAAAAACTGATCGAAAAATACCGCTGCGTAAAAGATACGGAAGGAATGTCTCCTGCTA
AGGTATATAAGCTGGTGGGAGAAAATGAAAACCTATATTTAAAAATGACGGACAGCCGGTATAAAGGGAC
CACCTATGATGTGGAACGGGAAAAGGACATGATGCTATGGCTGGAAGGAAAGCTGCCTGTTCCAAAGGTC
CTGCACTTTGAACGGCATGATGGCTGGAGCAATCTGCTCATGAGTGAGGCCGATGGCGTCCTTTGCTCGG
AAGAGTATGAAGATGAACAAAGCCCTGAAAAGATTATCGAGCTGTATGCGGAGTGCATCAGGCTCTTTCA
CTCCATCGACATATCGGATTGTCCCTATACGAATAGCTTAGACAGCCGCTTAGCCGAATTGGATTACTTA
CTGAATAACGATCTGGCCGATGTGGATTGCGAAAACTGGGAAGAAGACACTCCATTTAAAGATCCGCGCG
AGCTGTATGATTTTTTAAAGACGGAAAAGCCCGAAGAGGAACTTGTCTTTTCCCACGGCGACCTGGGAGA
CAGCAACATCTTTGTGAAAGATGGCAAAGTAAGTGGCTTTATTGATCTTGGGAGAAGCGGCAGGGCGGAC
AAGTGGTATGACATTGCCTTCTGCGTCCGGTCGATCAGGGAGGATATCGGGGAAGAACAGTATGTCGAGC
TATTTTTTGACTTACTGGGGATCAAGCCTGATTGGGAGAAAATAAAATATTATATTTTACTGGATGAATT
GTTTTAGGACGTCGCCGGCGGCATCAAATAAAACGAAAGGCTCAGTCGAAAGACTGGGCCTTTCGTTTTA
TCTGTTGTTTGTCGGTGAACGCTCTCCTGAGTAGGACAAATCCTCGAGaatatcaaattacgccccgccc
tgccactcatcgcagtactgttgtaattcattaagcattctgccgacatggaagccatcacaaacggcat
gatgaacctgaatcgccagcggcatcagcaccttgtcgccttgcgtataatatttgcccatggtgaaaac
gggggcgaagaagttgtccatattggccacgtttaaatcaaaactggtgaaactcacccagggattggct
gacacgaaaaacatattctcaataaaccctttagggaaataggccaggttttcaccgtaacacgccacat
cttgcgaatatatgtgtagaaactgccggaaatcgtcgtggtattcactccagagcgatgaaaacgtttc
agtttgctcatggaaaacggtgtaacaagggtgaacactatcccatatcaccagctcaccgtctttcatt

gccatacgaaattccggatgagcattcatcaggcgggcaagaatgtgaataaaggccggataaaacttgt
gcttatttttctttacggtctttaaaaaggccgtaatatccagctgaacggtctggttataggtacattg
agcaactgactgaaatgcctcaaaatgttctttacgatgccattgggatatatcaacggtggtatatcca
gtgatttttttctccattttagcttccttagctcctgaaaatctcgataactcaaaaaatacgcccggta
gtgatcttatttcattatggtgaaagttggaacctcttacgtgcccgatcaactcgagtgccacctgacg
tctaagaaaccattattatcatgacattaacctataaaaataggcgtatcacgaggcagaatttcagata
aaaaaaatccttagctttcgctaaggatgatttctggaattcgcggccgcttctaga

>pMB05(14,127bp)

ggtctcactatatctctatttaagacccactttcacatttaagttgtttttctaatccgcatatgatcaa
ttcaaggccgaataagaaggctggctctgcaccttggtgatcaaataattcgatagcttgtcgtaataat
ggcggcatactatcagtagtaggtgtttccctttcttctttagcgacttgatgctcttgatcttccaata
cgcaacctaaagtaaaatgccccacagcgctgagtgcatataatgcattctctagtgaaaaaccttgttg
gcataaaaaggctaattgattttcgagagtttcatactgtttttctgtaggccgtgtacctaaatgtact
tttgctccatcgcgatgacttagtaaagcacatctaaaacttttagcgttattacgtaaaaaatcttgcc
agctttccccttctaaagggcaaaagtgagtatggtgcctatctaacatctcaatggctaaggcgtcgag
caaagcccgcttatttttacatgccaatacaatgtaggctgctctacacctagcttctgggcgagttta
cgggttgttaaaccttcgattccgacctcattaagcagctctaatgcgctgttaatcactttactttat
ctaatctagacatcattaattcctaattttgttgacactctatcgttgatagagttattttaccactcc
ctatcagtgatagagaaaagaattcaaaagatctgtactttaactttaagaaggagatatataaaATGGA
CTACAAAGACCATGACGGTGATTATAAAGATCATGACATCGATTACAAGGATGACGATGACAAGCTCcat
atggataagaaatactcaataggcttagctatcggcacaaatagcgtcggatgggcggtgatcactgatg
aatataaggttccgtctaaaaagttcaaggttctgggaaatacagaccgccacagtatcaaaaaaaatct
tataggggctcttttatttgacagtggagagacagcggaagcgactcgtcttaaacggacagctcgtaga
aggtatacacgtcggaagaatcgtatttgttatctacaggagatttttcaaatgagatggcgaaagtag
atgatagtttctttcatcgacttgaagagtcttttttggtggaagaagacaagaagcatgaacgtcatcc
tatttttggaaatatagtagatgaagttgcttatcatgagaaatatccaactatctatcatctgcgaaaa
aaattggtagattctactgataaagcggatttgcgcttaatctatttggccttagcgcatatgattaagt
ttcgtggtcatttttttgattgagggagatttaaatcctgataatagtgatgtggacaaactatttatcca
gttggtacaaacctacaatcaattatttgaagaaaaccctattaacgcaagtggagtagatgctaaagcg
attctttctgcacgattgagtaaatcaagacgattagaaaatctcattgctcagctccccggtgagaaga
aaaatggcttatttgggaatctcattgctttgtcattgggtttgacccctaattttaaatcaaattttga
tttggcagaagatgctaaattacagctttcaaaagatacttacgatgatgatttagataatttattggcg
caaattggagatcaatatgctgatttgtttttggcagctaagaatttatcagatgctattttactttcag
atatcctaagagtaaatactgaaataactaaggctcccctatcagcttcaatgattaaacgctacgatga
acatcatcaagacttgactcttttaaaagctttagttcgacaacaacttccagaaaagtataaagaaatc
ttttttgatcaatcaaaaaacggatatgcaggttatattgatgggggagctagccaagaagaattttata
aatttatcaaaccaattttagaaaaaatggatggtactgaggaattattggtgaaactaaatcgtgaaga
tttgctgcgcaagcaacggacctttgacaacggctctattccccatcaaattcacttgggtgagctgcat
gctattttgagaagacaagaagacttttatccatttttaaaagacaatcgtgagaagattgaaaaaatct
tgacttttcgaattccttattatgttggtccattggcgcgtggcaatagtcgttttgcatggatgactcg
gaagtctgaagaaacaattaccccatggaattttgaagaagttgtcgataaaggtgcttcagctcaatca
tttattgaacgcatgacaaactttgataaaaatcttccaaatgaaaaagtactaccaaaacatagtttgc
tttatgagtattttacggtttataacgaattgacaaaggtcaaatatgttactgaaggaatgcgaaaacc
agcatttctttcaggtgaacagaagaaagccattgttgatttactcttcaaaacaaatcgaaaagtaacc
gttaagcaattaaaagaagattatttcaaaaaaatagaatgttttgatagtgttgaaatttcaggagttg
aagatagatttaatgcttcattaggtacctaccatgatttgctaaaaattattaaagataaagattttt
ggataatgaagaaaatgaagatatcttagaggatattgttttaacattgaccttatttgaagatagggag

```
atgattgaggaaagacttaaaacatatgctcacctctttgatgataaggtgatgaaacagcttaaacgtc
gccgttatactggttggggacgtttgtctcgaaaattgattaatggtattagggataagcaatctggcaa
aacaatattagatttttgaaatcagatggttttgccaatcgcaattttatgcagctgatccatgatgat
agtttgacatttaaagaagacattcaaaaagcacaagtgtctggacaaggcgatagtttacatgaacata
ttgcaaatttagctggtagccctgctattaaaaaaggtattttacagactgtaaaagttgttgatgaatt
ggtcaaagtaatggggcggcataagccagaaaatatcgttattgaaatggcacgtgaaaatcagacaact
caaaagggccagaaaaattcgcgagagcgtatgaaacgaatcgaagaaggtatcaaagaattaggaagtc
agattcttaaagagcatcctgttgaaaatactcaattgcaaaatgaaaagctctatctctattatctcca
aaatggaagagacatgtatgtggaccaagaattagatattaatcgtttaagtgattatgatgtcgatgcc
attgttccacaaagtttccttaaagacgattcaatagacaataaggtcttaacgcgttctgataaaaatc
gtggtaaatcggataacgttccaagtgaagaagtagtcaaaaagatgaaaaactattggagacaacttct
aaacgccaagttaatcactcaacgtaagtttgataatttaacgaaagctgaacgtggaggtttgagtgaa
cttgataaagctggttttatcaaacgccaattggttgaaactcgccaaatcactaagcatgtggcacaaa
ttttggatagtcgcatgaatactaaatacgatgaaaatgataaacttattcgagaggttaaagtgattac
cttaaaatctaaattagtttctgacttccgaaaagatttccaattctataaagtacgtgagattaacaat
taccatcatgcccatgatgcgtatctaaatgccgtcgttggaactgctttgattaagaaatatccaaaac
ttgaatcggagtttgtctatggtgattataaagtttatgatgttcgtaaaatgattgctaagtctgagca
agaaataggcaaagcaaccgcaaaatatttcttttactctaatatcatgaacttcttcaaaacagaaatt
acacttgcaaatggagagattcgcaaacgccctctaatcgaaactaatggggaaactggagaaattgtct
gggataaagggcgagattttgccacagtgcgcaaagtattgtccatgccccaagtcaatattgtcaagaa
aacagaagtacagacaggcggattctccaaggagtcaattttaccaaaaagaaattcggacaagcttatt
gctcgtaaaaaagactgggatccaaaaaaatatggtggttttgatagtccaacggtagcttattcagtcc
tagtggttgctaaggtggaaaaagggaaatcgaagaagttaaaatccgttaaagagttactagggatcac
aattatggaaagaagttcctttgaaaaaaatccgattgacttttttagaagctaaaggatataaggaagtt
aaaaaagacttaatcattaaactacctaaatatagtcttttttgagttagaaaacggtcgtaaacggatgc
tggctagtgccggagaattacaaaaaggaaatgagctggctctgccaagcaaatatgtgaatttttttata
tttagctagtcattatgaaaagttgaagggtagtccagaagataacgaacaaaaacaattgtttgtggag
cagcataagcattatttagatgagattattgagcaaatcagtgaattttctaagcgtgttattttagcag
atgccaatttagataaagttcttagtgcatataacaaacatagagacaaaccaatacgtgaacaagcaga
aaatattattcatttatttacgttgacgaatcttggagctcccgctgcttttaaatattttgatacaaca
attgatcgtaaacgatatacgtctacaaaagaagttttagatgccactcttatccatcaatccatcactg
gtctttatgaaacacgcattgatttgagtcagctaggaggtgactaactcgatcacactggctcaccttc
gggtgggcctttctgcgtttatatgtttgccctatcGATCTTTGACAGCTAGCTCAGTCCTAGGTATAAT
ACtagtGTACGCGCCTGGTATGGCAGGTTTTAGAGCTAGAAATAGCAAGTTAAAATAAGGCTAGTCCGTT
ATCAACTTGAAAAAGTGGCACCGAGTCGGTGCTTTTTTTGAAGCTTtgttccggctatttgatggctagc
tcagtccttggtattatgctagcgtactttaactttaagaaggagatatacatatggtgagcaagggcga
ggaggataacatggccatcatcaaggagttcatgcgcttcaaggtgcacatggagggctccgtgaacggc
cacgagttcgagatcgagggcgagggcgagggccgcccctacgagggcacccagaccgccaagctgaagg
tgaccaagggtggcccctgcccttcgcctgggacatcctgtcccctcagttcatgtacggctccaaggc
ctacgtgaagcaccccgccgacatccccgactacttgaagctgtccttccccgagggcttcaagtgggag
cgcgtgatgaacttcgaggacggcggcgtggtgaccgtgacccaggactcctccttgcaggacggcgagt
tcatctacaaggtgaagctgcgcggcaccaacttcccctccgacggccccgtaatgcagaagaagaccat
gggctgggaggcctcctccgagcggatgtaccccgaggacggcgccctgaagggcgagatcaagcagagg
ctgaagctgaaggacggcggccactacgacgctgaggtcaagaccacctacaaggccaagaagcccgtgc
agctgcccggcgcctacaacgtcaacatcaagttggacatcacctcccacaacgaggactacaccatcgt
ggaacagtacgaacgcgccgagggccgccactccaccggcggcatggacgagctgtacaagtaaggatcc
tcgatcacactggctcaccttcgggtgggcctttctgcgtttatatgttgaagctatttgatggctagct
cagtccttggtattatgctagcgtactttaactttaagaaggagatatacatatgcgtaaaggagaagaa
```

cttttcactggagttgtcccaattcttgttgaattagatggtgatgttaatgggcacaaattttctgtca
gtggagagggtgaaggtgatgcaacatacggaaaacttacccttaaatttatttgcactactggaaaact
acctgttccatggccaacacttgtcactactttcggttatggtgttcaatgctttgcgagatacccagat
catatgaaacagcatgactttttcaagagtgccatgcccgaaggttatgtacaggaaagaactatatttt
tcaaagatgacgggaactacaagacacgtgctgaagtcaagtttgaaggtgatacccttgttaatagaat
cgagttaaaaggtattgattttaaagaagatggaaacattcttggacacaaattggaatacaactataac
tcacacaatgtatacatcatggcagacaaacaaaagaatggaatcaaagttaacttcaaaattagacaca
acattgaagatggaagcgttcaactagcagaccattatcaacaaaatactccaattggcgatggccctgt
ccttttaccagacaaccattacctgtccacacaatctgccctttcgaaagatcccaacgaaaagagagat
cacatggtccttcttgagtttgtaacagctgctgggattacacatggcatggatgaactatacaaataat
cgatcacactggctcaccttcgggtgggcctttctgcgtttatatgttcttcctatcGATCTTTGACAGC
TAGCTCAGTCCTAGGTATAATACtagtGTACGCGCCTGGTATGGCAGGTTTTAGAGCTAGAAATAGCAAG
TTAAAATAAGGCTAGTCCGTTATCAACTTGAAAAAGTGGCACCGAGTCGGTGCTTTTTTTGAAGCTTtgt
tTTAGgtacagagaccctgcaGTTGCAAGACAATATCGACCGcttgcagcttgataaagtgatcaaagag
ggacgagcagctacagcactgctaagggagatcggagagccactgccgccaccagtgaaagtgaaaacag
gttggtttagtgctgattattacggctttgaggacatgctcacacgttgggataatgatcaatctagcct
ttccatttatcacagagagataaaagaggagcaacaggaacagcaacgaaaacaacagcagatagaaaga
gaaactaaacaaaaacaggaagatgacaaaaagaaacggcaggactatgaaagatatttatcaaaatacg
gctatgtcaaagatgctgagtatgaccatatcagcgaccatgttgcttcagacatttccatgtttacacg
tcttcaggcgcaggcatgggcaagtaacgatatgcaagagtacataaggtgttcaaagcaaaaatacgag
cagatcagcgacagaatagcctatgagcgtgacttgaaaaaaatcgggcaattgagccgtatcctggata
aagacagacaggcacttgctgatattttaccgcagttgatgccttactatgagcagatgcaacaggctgt
taataaacggaaaattttacttgagaatgagcgacagccatcatttaaaccacgttatgaccaagaacag
cctaagccgaaaaaagacaatgacctaacttttaatgtgttacaatagcattcaaatgtaacacattgg
agtgaaaaacatgacagaagcgacttttacctttcgggtagaccatgacctaaagcaagaattttcaagc
cttgctaagactgttgatcggtcgggggcgcagctaatccgtgacttcatgcgtgactttgttaagaagc
aacaggaagccgcagactatgacaaatggttcaagcagcaggtacagatcggcttaaatgaagccaatgc
aggcaagttgattccacatgaggacgtaaaggcagagtttgccgctagacgtgctgcaacattggcgaaa
ctggcagcgaagaatgaagattgagtggactgaaacagctcgccaagatcgtagaaatatctatgattat
cttgaagaacgcaaccctatagcagctattgaaattgatgatttaattgaagaaaagacagatttacttg
ttgataatcgactgatgggcgcacaggcagacagaaagatactagggagttagtgatacatccgcatta
tgtggttgtatatgacatcactgatataatacggatactcagagtgctacacacatcgcaggagtggtca
tgacttactcatgtactttggattatttagtgttataaaatcctgatttataaattttttttgttaaaaa
agataaaagccccttgcaattgcttggggctttaccgtaatttatggggtacagatcttcgatactgaca
tatcggcaatcgaaagcattaaggtttgacgaccgctaatgatttcaccacagggcttaatgtacctgt
cttaaattctaaggttttaactcgctttgtcaagcatagaccccaaaaatttagccaatgtctgtaactc
aatctgtccatgtgtgggtgatgaggtacagtgacgctagcacacatcggaaaaacgctattactagggg
aactgaacagagtagcggacgcaatgagtagtcatttaattggcggttatgagcgtgttcaggcggtgct
atcaatcgtaatcataacagtggcagcttgatacagtgatgtcatccctgatgcgaaagcgaccgaccga
cggtacatcgaatgggaatactttagggtgattttaagaatcgctctagggtgagtatttcccattcag
ctctgctccctccctctggtactttaatcaaaagcactactaaacatatgtttttaaataaaaaatattg
atatagagataatattagtaagaataattaaacaattgaatatagataaatcattgttaaataaagatta
attattaaaatgaatgtatacttatatataaatcaatgatttaaaatatttgataaagaaaacttttcaa
aaaaaatataattgagattgtgtcatttcggtcaattcttaatatgttccacgcaagttttagctatggt
gctaaacagaaatttgctgaaaaagaacttttcactgaactggttaaaatgtaagcagcctgagagccgc
caaaaattttaaaaacaaaccgccttaatcatcttcaaaaaatacctctaaaacctcaccatttgcgttt
taagacccatatttcatcctgcccttatgttcccatgctgatagctataaagtgtctgtaatcgcttcct
atgacgttctaggctgttgataacttttggaacaacgcaaaatgttaaaatccgatcatttttaaccta

gttattttcgttacagggtaactttggtagtattatttcaatatttagttgttacaggataacaaaatat
atgttacagggtaattaatttagcgttacaggataactatagttacagggtaacgttaaatagttatcct
gtaatttaaaaatagttgcaggatagcataaattttacaaataaattttataaaaagttatcctgtaac
taaaaacaatgttatcctgtaactatgatatactatatgggagttaaagacatgaaagacccgaacgatc
agaaaacacaggacatgctaaaagagccgccaaaaaccaatgccgaacgacaaaaagcatatcgtgaaaa
acgcaaaagccttgatagtcaacgtttggaggtgtttatagataagggtgtgtcggatatgcttgcggac
atggtgggcgcagcaggggagagccaaaaagccattttgaccgcattgattgagaaagagtataagcggc
tgtatgcagtgaaaaaatagcaagctaaaaaagtacattgattcaaaaagtaatgcagataaaaaagaaa
ccctcgatttatgagggtttttataactactaatgcctatgaaaataagtgcttatcattttctctaat
gtacccaataaaatcggggttttgttccacaagtttcttgatgtcattatcatgtttcttcagtaagtaa
gccccgatcaggatcttgcgttttgcatcgtctgaccttgcctgtttttgctctcgacctttcaccacag
cgtcagctagatcctttcttgccttttcctgacgttccaattttcctttcttcatcgagcatttgacc
caattccatagctgtttttttgaccattttaatgcctgtgtctcaacctattttccgaagtatgaacca
tcatcaatgtgaaaagtgttctacttcaaaaactcgacttacatcaagatccgagcgcagcgagcaaaat
caaaaaacaaagtcaaagccttattgctcttgcttttgcttttagcctcgcagagttcccgaaggcgca
cttacgcaaaattttgctacgccaaatttgcaagtacggtcagggaaaccccgacaccccaaccgccc
aaaacttggggcggctgttaataaacaggtgatgaaaatggcaatttaccattgtgaaatgcagaacatt
tcgaggtcagatggtcgctcaatcgtggcatgtgcagcataccgagcaggcgaaaaattgtactgtgata
cgtacggaaaagagcaggactacaccaaaaaaacaggcattgaatacacccaattttgccccaactgg
ggcaagtcctgacatgttagatcgtcaaaccctatggaatcggtagagcaatccgaactaaaaaagaac
ggtgacatcaaacaggaggcaagattagcaaaggaagtagagatagcattgccgcatgaactggataaga
cacagcgtcaggcacttgttaccgagttgtgccagtccttagttaaagcctatggagtggcggtggacgt
agcgatccatgcccctcatgtgcatggggaaggagaaagaaacctcacgcccacataagacacaGGCGT
CAGGCAACTTGTTAACTGCAgtccggcaaaaaagggcaaggtgtcaccaccctgccctttttctttaaaa
ccgaaaagattacttcgcgttatgcaggcttcctcgctcactgactcgctgcgctcggtcgttcggctgc
ggcgagcggtatcagctcactcaaaggcggtaatacggttatccacagaatcaggggataacgcaggaaa
gaacatgtgagcaaaaggccagcaaaaggccaggaaccgtaaaaaggccgcgttgctggcgtttttccac
aggctccgcccccctgacgagcatcacaaaaatcgacgctcaagtcagaggtggcgaaacccgacaggac
tataaagataccaggcgtttccccctggaagctccctcgtgcgctctcctgttccgaccctgccgcttac
cggatacctgtccgcctttctcccttcgggaagcgtggcgctttctcatagctcacgctgtaggtatctc
agttcggtgtaggtcgttcgctccaagctgggctgtgtgcacgaaccccccgttcagcccgaccgctgcg
ccttatccggtaactatcgtcttgagtccaacccggtaagacacgacttatcgccactggcagcagccac
tggtaacaggattagcagagcgaggtatgtaggcggtgctacagagttcttgaagtggtggcctaactac
ggctacactagaagaacagtatttggtatctgcgctctgctgaagccagttaccttcggaaaaagagttg
gtagctcttgatccggcaaacaaaccaccgctggtagcggtggtttttttgtttgcaagcagcagattac
gcgcagaaaaaaaggatctcaagaagatcctttgatcttttctacggggtctgacgctcagtggaacgaa
aactcacgttaagggattttggtcatgagattatcaaaaaggatcttcacctagatccttttaaattaaa
aatgaagttttaaatcaatctaaagtatatatgagtaaacttggtctgacagctcgaggcttggattctc
accaataaaaaacgcccggcggcaaccgagcgttctgaacaaatccagatggagttctgaggtcattact
ggatctatcaacaggagtccaagcgagctcgatattCTCGAGAAGCTGGGGATCCGTTTGATTTTTAATG
GATAATGTGATATAATCTTTAAATACTGTAGAAAAGAGGAAGGAAATAATAAATGGCTAAAATGAGAATA
TCACCGGAATTGAAAAAACTGATCGAAAAATACCGCTGCGTAAAAGATACGGAAGGAATGTCTCCTGCTA
AGGTATATAAGCTGGTGGGAGAAAATGAAAACCTATATTTAAAAATGACGGACAGCCGGTATAAAGGGAC
CACCTATGATGTGGAACGGGAAAAGGACATGATGCTATGGCTGGAAGGAAAGCTGCCTGTTCCAAAGGTC
CTGCACTTTGAACGGCATGATGGCTGGAGCAATCTGCTCATGAGTGAGGCCGATGGCGTCCTTTGCTCGG
AAGAGTATGAAGATGAACAAAGCCCTGAAAAGATTATCGAGCTGTATGCGGAGTGCATCAGGCTCTTTCA
CTCCATCGACATATCGGATTGTCCCTATACGAATAGCTTAGACAGCCGCTTAGCCGAATTGGATTACTTA
CTGAATAACGATCTGGCCGATGTGGATTGCGAAAACTGGGAAGAAGACACTCCATTTAAAGATCCGCGCG

```
AGCTGTATGATTTTTTAAAGACGGAAAAGCCCGAAGAGGAACTTGTCTTTTCCCACGGCGACCTGGGAGA
CAGCAACATCTTTGTGAAAGATGGCAAAGTAAGTGGCTTTATTGATCTTGGGAGAAGCGGCAGGGCGGAC
AAGTGGTATGACATTGCCTTCTGCGTCCGGTCGATCAGGGAGGATATCGGGGAAGAACAGTATGTCGAGC
TATTTTTTGACTTACTGGGGATCAAGCCTGATTGGGAGAAAATAAAATATTATATTTTACTGGATGAATT
GTTTTAGGACGTCGCCGGCGGCATCAAATAAAACGAAAGGCTCAGTCGAAAGACTGGGCCTTTCGTTTTA
TCTGTTGTTTGTCGGTGAACGCTCTCCTGAGTAGGACAAATCCTCGAGaatatcaaattacgccccgccc
tgccactcatcgcagtactgttgtaattcattaagcattctgccgacatggaagccatcacaaacggcat
gatgaacctgaatcgccagcggcatcagcaccttgtcgccttgcgtataatatttgcccatggtgaaaac
gggggcgaagaagttgtccatattggccacgtttaaatcaaaactggtgaaactcacccagggattggct
gacacgaaaaacatattctcaataaaccctttagggaaataggccaggttttcaccgtaacacgccacat
cttgcgaatatatgtgtagaaactgccggaaatcgtcgtggtattcactccagagcgatgaaaacgtttc
agtttgctcatggaaaacggtgtaacaagggtgaacactatcccatatcaccagctcaccgtctttcatt
gccatacgaaattccggatgagcattcatcaggcgggcaagaatgtgaataaaggccggataaaacttgt
gcttatttttctttacggtctttaaaaaggccgtaatatccagctgaacggtctggttataggtacattg
agcaactgactgaaatgcctcaaaatgttctttacgatgccattgggatatatcaacggtggtatatcca
gtgatttttttctccattttagcttccttagctcctgaaaatctcgataactcaaaaaatacgcccggta
gtgatcttatttcattatggtgaaagttggaacctcttacgtgcccgatcaactcgagtgccacctgacg
tctaagaaaccattattatcatgacattaacctataaaaataggcgtatcacgaggcagaatttcagata
aaaaaaatccttagctttcgctaaggatgatttctggaattcgcggccgcttctaga
```

Sequence alignment of pMB1-A-strong_*gfp* extracted from *A. baumannii* at different timepoints

```
day0            1    AAAAAAAATATAATTGAGATTGTGTCATTTCGGTCAATTCTTAATATGTTCCACGCAAGT
day5_carb       1    AAAAAAAATATAATTGAGATTGTGTCATTTCGGTCAATTCTTAATATGTTCCACGCAAGT
day5_nocarb     1    AAAAAAAATATAATTGAGATTGTGTCATTTCGGTCAATTCTTAATATGTTCCACGCAAGT

day0            61   TTTAGCTATGGTGCTAAACAGAAATTTGCTGAAAAAGAACTTTTCACTGAACTGGTTAAA
day5_carb       61   TTTAGCTATGGTGCTAAACAGAAATTTGCTGAAAAAGAACTTTTCACTGAACTGGTTAAA
day5_nocarb     61   TTTAGCTATGGTGCTAAACAGAAATTTGCTGAAAAAGAACTTTTCACTGAACTGGTTAAA

day0            121  ATGTAAGCAGCCTGAGAGCCGCCAAAAATTTTAAAAACAAACCGCCTTAATCATCTTCAA
day5_carb       121  ATGTAAGCAGCCTGAGAGCCGCCAAAAATTTTAAAAACAAACCGCCTTAATCATCTTCAA
day5_nocarb     121  ATGTAAGCAGCCTGAGAGCCGCCAAAAATTTTAAAAACAAACCGCCTTAATCATCTTCAA

day0            181  AAAATACCTCTAAAACCTCACCATTTGCGTTTTAAGACCCATATTTCATCCTGCCCTTAT
day5_carb       181  AAAATACCTCTAAAACCTCACCATTTGCGTTTTAAGACCCATATTTCATCCTGCCCTTAT
day5_nocarb     181  AAAATACCTCTAAAACCTCACCATTTGCGTTTTAAGACCCATATTTCATCCTGCCCTTAT

day0            241  GTTCCCATGCTGATAGCTATAAAGTGTCTGTAATCGCTTCCTATGACGTTCTAGGCTGTT
day5_carb       241  GTTCCCATGCTGATAGCTATAAAGTGTCTGTAATCGCTTCCTATGACGTTCTAGGCTGTT
day5_nocarb     241  GTTCCCATGCTGATAGCTATAAAGTGTCTGTAATCGCTTCCTATGACGTTCTAGGCTGTT

day0            301  GATAACTTTTGGAACAACGCAAAATGTTAAAATCCGATCATTTTTTAACCTAGTTATTTT
day5_carb       301  GATAACTTTTGGAACAACGCAAAATGTTAAAATCCGATCATTTTTTAACCTAGTTATTTT
day5_nocarb     301  GATAACTTTTGGAACAACGCAAAATGTTAAAATCCGATCATTTTTTAACCTAGTTATTTT

day0            361  CGTTACAGGGTAACTTTGGTAGTATTATTTCAATATTTAGTTGTTACAGGATAACAAAAT
day5_carb       361  CGTTACAGGGTAACTTTGGTAGTATTATTTCAATATTTAGTTGTTACAGGATAACAAAAT
day5_nocarb     361  CGTTACAGGGTAACTTTGGTAGTATTATTTCAATATTTAGTTGTTACAGGATAACAAAAT

day0            421  ATATGTTACAGGGTAATTAATTTAGCGTTACAGGATAACTATAGTTACAGGGTAACGTTA
day5_carb       421  ATATGTTACAGGGTAATTAATTTAGCGTTACAGGATAACTATAGTTACAGGGTAACGTTA
```

```
day5_nocarb     421     ATATGTTACAGGGTAATTAATTTAGCGTTACAGGATAACTATAGTTACAGGGTAACGTTA

day0            481     AATAGTTATCCTGTAATTTAAAAATAGTTGCAGGATAGCATAAATTTTTACAAATAAATT
day5_carb       481     AATAGTTATCCTGTAATTTAAAAATAGTTGCAGGATAGCATAAATTTTTACAAATAAATT
day5_nocarb     481     AATAGTTATCCTGTAATTTAAAAATAGTTGCAGGATAGCATAAATTTTTACAAATAAATT

day0            541     TTATAAAAAGTTATCCTGTAACTAAAAACAATGTTATCCTGTAACTATGATATACTATAT
day5_carb       541     TTATAAAAAGTTATCCTGTAACTAAAAACAATGTTATCCTGTAACTATGATATACTATAT
day5_nocarb     541     TTATAAAAAGTTATCCTGTAACTAAAAACAATGTTATCCTGTAACTATGATATACTATAT

day0            601     GGGAGTTAAAGACATGAAAGACCCGAACGATCAGAAAACACAGGACATGCTAAAAGAGCC
day5_carb       601     GGGAGTTAAAGACATGAAAGACCCGAACGATCAGAAAACACAGGACATGCTAAAAGAGCC
day5_nocarb     601     GGGAGTTAAAGACATGAAAGACCCGAACGATCAGAAAACACAGGACATGCTAAAAGAGCC

day0            661     GCCAAAAACCAATGCCGAACGACAAAAAGCATATCGTGAAAAACGCAAAAGCCTTGATAG
day5_carb       661     GCCAAAAACCAATGCCGAACGACAAAAAGCATATCGTGAAAAACGCAAAAGCCTTGATAG
day5_nocarb     661     GCCAAAAACCAATGCCGAACGACAAAAAGCATATCGTGAAAAACGCAAAAGCCTTGATAG

day0            721     TCAACGTTTGGAGGTGTTTATAGATAAGGGTGTGTCGGATATGCTTGCGGACATGGTGGG
day5_carb       721     TCAACGTTTGGAGGTGTTTATAGATAAGGGTGTGTCGGATATGCTTGCGGACATGGTGGG
day5_nocarb     721     TCAACGTTTGGAGGTGTTTATAGATAAGGGTGTGTCGGATATGCTTGCGGACATGGTGGG

day0            781     CGCAGCAGGGGAGAGCCAAAAAGCCATTTTGACCGCATTGATTGAGAAAGAGTATAAGCG
day5_carb       781     CGCAGCAGGGGAGAGCCAAAAAGCCATTTTGACCGCATTGATTGAGAAAGAGTATAAGCG
day5_nocarb     781     CGCAGCAGGGGAGAGCCAAAAAGCCATTTTGACCGCATTGATTGAGAAAGAGTATAAGCG

day0            841     GCTGTATGCAGTGAAAAATAGCAAGCTAAAAAAGTACATTGATTCAAAAAGTAATGCAGA
day5_carb       841     GCTGTATGCAGTGAAAAATAGCAAGCTAAAAAAGTACATTGATTCAAAAAGTAATGCAGA
day5_nocarb     841     GCTGTATGCAGTGAAAAATAGCAAGCTAAAAAAGTACATTGATTCAAAAAGTAATGCAGA

day0            901     TAAAAAAGAAACCCTCGATTTATGAGGGTTTTTTATAACTACTAATGCCTATGAAAATAA
day5_carb       901     TAAAAAAGAAACCCTCGATTTATGAGGGTTTTTTATAACTACTAATGCCTATGAAAATAA
day5_nocarb     901     TAAAAAAGAAACCCTCGATTTATGAGGGTTTTTTATAACTACTAATGCCTATGAAAATAA

day0            961     GTGCTTATCATTTTCTCTAATGTACCCAATAAAATCGGGGTTTTGTTCCACAAGTTTCTT
day5_carb       961     GTGCTTATCATTTTCTCTAATGTACCCAATAAAATCGGGGTTTTGTTCCACAAGTTTCTT
day5_nocarb     961     GTGCTTATCATTTTCTCTAATGTACCCAATAAAATCGGGGTTTTGTTCCACAAGTTTCTT

day0            1021    GATGTCATTATCATGTTTCTTCAGTAAGTAAGCCCCGATCAGGATCTTGCGTTTTGCATC
day5_carb       1021    GATGTCATTATCATGTTTCTTCAGTAAGTAAGCCCCGATCAGGATCTTGCGTTTTGCATC
day5_nocarb     1021    GATGTCATTATCATGTTTCTTCAGTAAGTAAGCCCCGATCAGGATCTTGCGTTTTGCATC

day0            1081    GTCTGACCTTGCCTGTTTTTGCTCTCGACCTTTCACCACAGCGTCAGCTAGATCCTTTCT
day5_carb       1081    GTCTGACCTTGCCTGTTTTTGCTCTCGACCTTTCACCACAGCGTCAGCTAGATCCTTTCT
day5_nocarb     1081    GTCTGACCTTGCCTGTTTTTGCTCTCGACCTTTCACCACAGCGTCAGCTAGATCCTTTCT

day0            1141    TGCCTTTTCCTGACGTTCCAATTTTTCCTTTTCTTCATCGAGCATTTGACCCAATTCCAT
day5_carb       1141    TGCCTTTTCCTGACGTTCCAATTTTTCCTTTTCTTCATCGAGCATTTGACCCAATTCCAT
day5_nocarb     1141    TGCCTTTTCCTGACGTTCCAATTTTTCCTTTTCTTCATCGAGCATTTGACCCAATTCCAT

day0            1201    AGCTGTTTTTTTGACCATTTTTAATGCCTGTGTCTCAACCTATTTTCCGAAGTATGAACC
day5_carb       1201    AGCTGTTTTTTTGACCATTTTTAATGCCTGTGTCTCAACCTATTTTCCGAAGTATGAACC
day5_nocarb     1201    AGCTGTTTTTTTGACCATTTTTAATGCCTGTGTCTCAACCTATTTTCCGAAGTATGAACC

day0            1261    ATCATCAATGTGAAAAGTGTTCTACTTCAAAAACTCGACTTACATCAAGATCCGAGCGCA
day5_carb       1261    ATCATCAATGTGAAAAGTGTTCTACTTCAAAAACTCGACTTACATCAAGATCCGAGCGCA
day5_nocarb     1261    ATCATCAATGTGAAAAGTGTTCTACTTCAAAAACTCGACTTACATCAAGATCCGAGCGCA
```

```
day0            1321    GCGAGCAAAATCAAAAAACAAAGTCAAAGCCTTATTGCTCTTGCTTTTGCTTTTAGCCTC
day5_carb       1321    GCGAGCAAAATCAAAAAACAAAGTCAAAGCCTTATTGCTCTTGCTTTTGCTTTTAGCCTC
day5_nocarb     1321    GCGAGCAAAATCAAAAAACAAAGTCAAAGCCTTATTGCTCTTGCTTTTGCTTTTAGCCTC

day0            1381    GCAGAGTTCCCGAAGGGCGCACTTACGCAAAATTTTTGCTACGCCAAATTTTGCAAGTAC
day5_carb       1381    GCAGAGTTCCCGAAGGGCGCACTTACGCAAAATTTTTGCTACGCCAAATTTTGCAAGTAC
day5_nocarb     1381    GCAGAGTTCCCGAAGGGCGCACTTACGCAAAATTTTTGCTACGCCAAATTTTGCAAGTAC

day0            1441    GGTCAGGGAAACCCCGACACCCCAACCGCCCAAAACTTGGGGCGGCTGTTAATAAACAGG
day5_carb       1441    GGTCAGGGAAACCCCGACACCCCAACCGCCCAAAACTTGGGGCGGCTGTTAATAAACAGG
day5_nocarb     1441    GGTCAGGGAAACCCCGACACCCCAACCGCCCAAAACTTGGGGCGGCTGTTAATAAACAGG

day0            1501    TGATGAAAATGGCAATTTACCATTGTGAAATGCAGAACATTTCGAGGTCAGATGGTCGCT
day5_carb       1501    TGATGAAAATGGCAATTTACCATTGTGAAATGCAGAACATTTCGAGGTCAGATGGTCGCT
day5_nocarb     1501    TGATGAAAATGGCAATTTACCATTGTGAAATGCAGAACATTTCGAGGTCAGATGGTCGCT

day0            1561    CAATCGTGGCATGTGCAGCATACCGAGCAGGCGAAAAATTGTACTGTGATACGTACGGAA
day5_carb       1561    CAATCGTGGCATGTGCAGCATACCGAGCAGGCGAAAAATTGTACTGTGATACGTACGGAA
day5_nocarb     1561    CAATCGTGGCATGTGCAGCATACCGAGCAGGCGAAAAATTGTACTGTGATACGTACGGAA

day0            1621    AAGAGCAGGACTACACCAAAAAAACAGGCATTGAATACACCCAAATTTTTGCCCCAACTG
day5_carb       1621    AAGAGCAGGACTACACCAAAAAAACAGGCATTGAATACACCCAAATTTTTGCCCCAACTG
day5_nocarb     1621    AAGAGCAGGACTACACCAAAAAAACAGGCATTGAATACACCCAAATTTTTGCCCCAACTG

day0            1681    GGGCAAGTCCTGACATGTTAGATCGTCAAACCCTATGGAATCGGGTAGAGCAATCCGAAC
day5_carb       1681    GGGCAAGTCCTGACATGTTAGATCGTCAAACCCTATGGAATCGGGTAGAGCAATCCGAAC
day5_nocarb     1681    GGGCAAGTCCTGACATGTTAGATCGTCAAACCCTATGGAATCGGGTAGAGCAATCCGAAC

day0            1741    TAAAAAAGAACGGTGACATCAAACAGGAGGCAAGATTAGCAAAGGAAGTAGAGATAGCAT
day5_carb       1741    TAAAAAAGAACGGTGACATCAAACAGGAGGCAAGATTAGCAAAGGAAGTAGAGATAGCAT
day5_nocarb     1741    TAAAAAAGAACGGTGACATCAAACAGGAGGCAAGATTAGCAAAGGAAGTAGAGATAGCAT

day0            1801    TGCCGCATGAACTGGATAAGACACAGCGTCAGGCAACTTGTTAACTGCAGCGTCTCTGGC
day5_carb       1801    TGCCGCATGAACTGGATAAGACACAGCGTCAGGCAACTTGTTAACTGCAGCGTCTCTGGC
day5_nocarb     1801    TGCCGCATGAACTGGATAAGACACAGCGTCAGGCAACTTGTTAACTGCAGCGTCTCTGGC

day0            1861    AAACATATAAACGCAGAAAGGCCCACCCGAAGGTGAGCCAGTGTGATCGATTATTTGTAT
day5_carb       1861    AAACATATAAACGCAGAAAGGCCCACCCGAAGGTGAGCCAGTGTGATCGATTATTTGTAT
day5_nocarb     1861    AAACATATAAACGCAGAAAGGCCCACCCGAAGGTGAGCCAGTGTGATCGATTATTTGTAT

day0            1921    AGTTCATCCATGCCATGTGTAATCCCAGCAGCTGTTACAAACTCAAGAAGGACCATGTGA
day5_carb       1921    AGTTCATCCATGCCATGTGTAATCCCAGCAGCTGTTACAAACTCAAGAAGGACCATGTGA
day5_nocarb     1921    AGTTCATCCATGCCATGTGTAATCCCAGCAGCTGTTACAAACTCAAGAAGGACCATGTGA

day0            1981    TCTCTCTTTTCGTTGGGATCTTTCGAAAGGGCAGATTGTGTGGACAGGTAATGGTTGTCT
day5_carb       1981    TCTCTCTTTTCGTTGGGATCTTTCGAAAGGGCAGATTGTGTGGACAGGTAATGGTTGTCT
day5_nocarb     1981    TCTCTCTTTTCGTTGGGATCTTTCGAAAGGGCAGATTGTGTGGACAGGTAATGGTTGTCT

day0            2041    GGTAAAAGGACAGGGCCATCGCCAATTGGAGTATTTTGTTGATAATGGTCTGCTAGTTGA
day5_carb       2041    GGTAAAAGGACAGGGCCATCGCCAATTGGAGTATTTTGTTGATAATGGTCTGCTAGTTGA
day5_nocarb     2041    GGTAAAAGGACAGGGCCATCGCCAATTGGAGTATTTTGTTGATAATGGTCTGCTAGTTGA

day0            2101    ACGCTTCCATCTTCAATGTTGTGTCTAATTTTGAAGTTAACTTTGATTCCATTCTTTTGT
day5_carb       2101    ACGCTTCCATCTTCAATGTTGTGTCTAATTTTGAAGTTAACTTTGATTCCATTCTTTTGT
day5_nocarb     2101    ACGCTTCCATCTTCAATGTTGTGTCTAATTTTGAAGTTAACTTTGATTCCATTCTTTTGT
```

```
day0                2161  TTGTCTGCCATGATGTATACATTGTGTGAGTTATAGTTGTATTCCAATTTGTGTCCAAGA
day5_carb           2161  TTGTCTGCCATGATGTATACATTGTGTGAGTTATAGTTGTATTCCAATTTGTGTCCAAGA
day5_nocarb         2161  TTGTCTGCCATGATGTATACATTGTGTGAGTTATAGTTGTATTCCAATTTGTGTCCAAGA

day0                2221  ATGTTTCCATCTTCTTTAAAATCAATACCTTTTAACTCGATTCTATTAACAAGGGTATCA
day5_carb           2221  ATGTTTCCATCTTCTTTAAAATCAATACCTTTTAACTCGATTCTATTAACAAGGGTATCA
day5_nocarb         2221  ATGTTTCCATCTTCTTTAAAATCAATACCTTTTAACTCGATTCTATTAACAAGGGTATCA

day0                2281  CCTTCAAACTTGACTTCAGCACGTGTCTTGTAGTTCCCGTCATCTTTGAAAAATATAGTT
day5_carb           2281  CCTTCAAACTTGACTTCAGCACGTGTCTTGTAGTTCCCGTCATCTTTGAAAAATATAGTT
day5_nocarb         2281  CCTTCAAACTTGACTTCAGCACGTGTCTTGTAGTTCCCGTCATCTTTGAAAAATATAGTT

day0                2341  CTTTCCTGTACATAACCTTCGGGCATGGCACTCTTGAAAAAGTCATGCTGTTTCATATGA
day5_carb           2341  CTTTCCTGTACATAACCTTCGGGCATGGCACTCTTGAAAAAGTCATGCTGTTTCATATGA
day5_nocarb         2341  CTTTCCTGTACATAACCTTCGGGCATGGCACTCTTGAAAAAGTCATGCTGTTTCATATGA

day0                2401  TCTGGGTATCTCGCAAAGCATTGAACACCATAACCGAAAGTAGTGACAAGTGTTGGCCAT
day5_carb           2401  TCTGGGTATCTCGCAAAGCATTGAACACCATAACCGAAAGTAGTGACAAGTGTTGGCCAT
day5_nocarb         2401  TCTGGGTATCTCGCAAAGCATTGAACACCATAACCGAAAGTAGTGACAAGTGTTGGCCAT

day0                2461  GGAACAGGTAGTTTTCCAGTAGTGCAAATAAATTTAAGGGTAAGTTTTCCGTATGTTGCA
day5_carb           2461  GGAACAGGTAGTTTTCCAGTAGTGCAAATAAATTTAAGGGTAAGTTTTCCGTATGTTGCA
day5_nocarb         2461  GGAACAGGTAGTTTTCCAGTAGTGCAAATAAATTTAAGGGTAAGTTTTCCGTATGTTGCA

day0                2521  TCACCTTCACCCTCTCCACTGACAGAAAATTTGTGCCCATTAACATCACCATCTAATTCA
day5_carb           2521  TCACCTTCACCCTCTCCACTGACAGAAAATTTGTGCCCATTAACATCACCATCTAATTCA
day5_nocarb         2521  TCACCTTCACCCTCTCCACTGACAGAAAATTTGTGCCCATTAACATCACCATCTAATTCA

day0                2581  ACAAGAATTGGGACAACTCCAGTGAAAAGTTCTTCTCCTTTACGCATATGTATATCTCCT
day5_carb           2581  ACAAGAATTGGGACAACTCCAGTGAAAAGTTCTTCTCCTTTACGCATATGTATATCTCCT
day5_nocarb         2581  ACAAGAATTGGGACAACTCCAGTGAAAAGTTCTTCTCCTTTACGCATATGTATATCTCCT

day0                2641  TCTTAAAGTTAAAGTACGCTAGCATAATACCAAGGACTGAGCTAGCCATCAAATAGAGAT
day5_carb           2641  TCTTAAAGTTAAAGTACGCTAGCATAATACCAAGGACTGAGCTAGCCATCAAATAGAGAT
day5_nocarb         2641  TCTTAAAGTTAAAGTACGCTAGCATAATACCAAGGACTGAGCTAGCCATCAAATAGAGAT

day0                2701  TGAGACGTTCTAGAAGCGGCCGCGAATTCCAGAAATCATCCTTAGCGAAAGCTAAGGATT
day5_carb           2701  TGAGACGTTCTAGAAGCGGCCGCGAATTCCAGAAATCATCCTTAGCGAAAGCTAAGGATT
day5_nocarb         2701  TGAGACGTTCTAGAAGCGGCCGCGAATTCCAGAAATCATCCTTAGCGAAAGCTAAGGATT

day0                2761  TTTTTTATCTGAAATTCTGCCTCGTGATACGCCTATTTTTATAGGTTAATGTCATGATAA
day5_carb           2761  TTTTTTATCTGAAATTCTGCCTCGTGATACGCCTATTTTTATAGGTTAATGTCATGATAA
day5_nocarb         2761  TTTTTTATCTGAAATTCTGCCTCGTGATACGCCTATTTTTATAGGTTAATGTCATGATAA

day0                2821  TAATGGTTTCTTAGACGTCAGGTGGCACTTTTCGGGGAAATGTGCGCGGAACCCCTATTT
day5_carb           2821  TAATGGTTTCTTAGACGTCAGGTGGCACTTTTCGGGGAAATGTGCGCGGAACCCCTATTT
day5_nocarb         2821  TAATGGTTTCTTAGACGTCAGGTGGCACTTTTCGGGGAAATGTGCGCGGAACCCCTATTT

day0                2881  GTTTATTTTTCTAAATACATTCAAATATGTATCCGCTCATGAGACAATAACCCTGATAAA
day5_carb           2881  GTTTATTTTTCTAAATACATTCAAATATGTATCCGCTCATGAGACAATAACCCTGATAAA
day5_nocarb         2881  GTTTATTTTTCTAAATACATTCAAATATGTATCCGCTCATGAGACAATAACCCTGATAAA

day0                2941  TGCTTCAATAATATTGAAAAAGGAAGAGTATGAGTATTCAACATTTCCGTGTCGCCCTTA
day5_carb           2941  TGCTTCAATAATATTGAAAAAGGAAGAGTATGAGTATTCAACATTTCCGTGTCGCCCTTA
day5_nocarb         2941  TGCTTCAATAATATTGAAAAAGGAAGAGTATGAGTATTCAACATTTCCGTGTCGCCCTTA

day0                3001  TTCCCTTTTTTGCGGCATTTTGCCTTCCTGTTTTTGCTCACCCAGAAACGCTGGTGAAAG
```

```
day5_carb        3001   TTCCCTTTTTTGCGGCATTTTGCCTTCCTGTTTTTGCTCACCCAGAAACGCTGGTGAAAG
day5_nocarb      3001   TTCCCTTTTTTGCGGCATTTTGCCTTCCTGTTTTTGCTCACCCAGAAACGCTGGTGAAAG

day0             3061   TAAAAGATGCTGAAGATCAGTTGGGTGCACGAGTGGGTTACATCGAACTGGATCTCAACA
day5_carb        3061   TAAAAGATGCTGAAGATCAGTTGGGTGCACGAGTGGGTTACATCGAACTGGATCTCAACA
day5_nocarb      3061   TAAAAGATGCTGAAGATCAGTTGGGTGCACGAGTGGGTTACATCGAACTGGATCTCAACA

day0             3121   GCGGTAAGATCCTTGAGAGTTTTCGCCCCGAAGAACGTTTTCCAATGATGAGCACTTTTA
day5_carb        3121   GCGGTAAGATCCTTGAGAGTTTTCGCCCCGAAGAACGTTTTCCAATGATGAGCACTTTTA
day5_nocarb      3121   GCGGTAAGATCCTTGAGAGTTTTCGCCCCGAAGAACGTTTTCCAATGATGAGCACTTTTA

day0             3181   AAGTTCTGCTATGTGGCGCGGTATTATCCCGTATTGACGCCGGGCAAGAGCAACTCGGTC
day5_carb        3181   AAGTTCTGCTATGTGGCGCGGTATTATCCCGTATTGACGCCGGGCAAGAGCAACTCGGTC
day5_nocarb      3181   AAGTTCTGCTATGTGGCGCGGTATTATCCCGTATTGACGCCGGGCAAGAGCAACTCGGTC

day0             3241   GCCGCATACACTATTCTCAGAATGACTTGGTTGAGTACTCACCAGTCACAGAAAAGCATC
day5_carb        3241   GCCGCATACACTATTCTCAGAATGACTTGGTTGAGTACTCACCAGTCACAGAAAAGCATC
day5_nocarb      3241   GCCGCATACACTATTCTCAGAATGACTTGGTTGAGTACTCACCAGTCACAGAAAAGCATC

day0             3301   TTACGGATGGCATGACAGTAAGAGAATTATGCAGTGCTGCCATAACCATGAGTGATAACA
day5_carb        3301   TTACGGATGGCATGACAGTAAGAGAATTATGCAGTGCTGCCATAACCATGAGTGATAACA
day5_nocarb      3301   TTACGGATGGCATGACAGTAAGAGAATTATGCAGTGCTGCCATAACCATGAGTGATAACA

day0             3361   CTGCGGCCAACTTACTTCTGACAACGATCGGAGGACCGAAGGAGCTAACCGCTTTTTTGC
day5_carb        3361   CTGCGGCCAACTTACTTCTGACAACGATCGGAGGACCGAAGGAGCTAACCGCTTTTTTGC
day5_nocarb      3361   CTGCGGCCAACTTACTTCTGACAACGATCGGAGGACCGAAGGAGCTAACCGCTTTTTTGC

day0             3421   ACAACATGGGGGATCATGTAACTCGCCTTGATCGTTGGGAACCGGAGCTGAATGAAGCCA
day5_carb        3421   ACAACATGGGGGATCATGTAACTCGCCTTGATCGTTGGGAACCGGAGCTGAATGAAGCCA
day5_nocarb      3421   ACAACATGGGGGATCATGTAACTCGCCTTGATCGTTGGGAACCGGAGCTGAATGAAGCCA

day0             3481   TACCAAACGACGAGCGTGACACCACGATGCCTGTAGCAATGGCAACAACGTTGCGCAAAC
day5_carb        3481   TACCAAACGACGAGCGTGACACCACGATGCCTGTAGCAATGGCAACAACGTTGCGCAAAC
day5_nocarb      3481   TACCAAACGACGAGCGTGACACCACGATGCCTGTAGCAATGGCAACAACGTTGCGCAAAC

day0             3541   TATTAACTGGCGAACTACTTACTCTAGCTTCCCGGCAACAATTAATAGACTGGATGGAGG
day5_carb        3541   TATTAACTGGCGAACTACTTACTCTAGCTTCCCGGCAACAATTAATAGACTGGATGGAGG
day5_nocarb      3541   TATTAACTGGCGAACTACTTACTCTAGCTTCCCGGCAACAATTAATAGACTGGATGGAGG

day0             3601   CGGATAAAGTTGCAGGACCACTTCTGCGCTCGGCCCTTCCGGCTGGCTGGTTTATTGCTG
day5_carb        3601   CGGATAAAGTTGCAGGACCACTTCTGCGCTCGGCCCTTCCGGCTGGCTGGTTTATTGCTG
day5_nocarb      3601   CGGATAAAGTTGCAGGACCACTTCTGCGCTCGGCCCTTCCGGCTGGCTGGTTTATTGCTG

day0             3661   ATAAATCTGGAGCCGGTGAGCGTGGCTCTCGCGGTATCATTGCAGCACTGGGGCCAGATG
day5_carb        3661   ATAAATCTGGAGCCGGTGAGCGTGGCTCTCGCGGTATCATTGCAGCACTGGGGCCAGATG
day5_nocarb      3661   ATAAATCTGGAGCCGGTGAGCGTGGCTCTCGCGGTATCATTGCAGCACTGGGGCCAGATG

day0             3721   GTAAGCCCTCCCGTATCGTAGTTATCTACACGACGGGGAGTCAGGCAACTATGGATGAAC
day5_carb        3721   GTAAGCCCTCCCGTATCGTAGTTATCTACACGACGGGGAGTCAGGCAACTATGGATGAAC
day5_nocarb      3721   GTAAGCCCTCCCGTATCGTAGTTATCTACACGACGGGGAGTCAGGCAACTATGGATGAAC

day0             3781   GAAATAGACAGATCGCTGAGATAGGTGCCTCACTGATTAAGCATTGGTAACTGTCAGACC
day5_carb        3781   GAAATAGACAGATCGCTGAGATAGGTGCCTCACTGATTAAGCATTGGTAACTGTCAGACC
day5_nocarb      3781   GAAATAGACAGATCGCTGAGATAGGTGCCTCACTGATTAAGCATTGGTAACTGTCAGACC

day0             3841   AAGTTTACTCATATATACTTTAGATTGATTTAAAACTTCATTTTTAATTTAAAAGGATCT
day5_carb        3841   AAGTTTACTCATATATACTTTAGATTGATTTAAAACTTCATTTTTAATTTAAAAGGATCT
```

```
day5_nocarb     3841    AAGTTTACTCATATATACTTTAGATTGATTTAAAACTTCATTTTTAATTTAAAAGGATCT

day0            3901    AGGTGAAGATCCTTTTTGATAATCTCATGACCAAAATCCCTTAACGTGAGTTTTCGTTCC
day5_carb       3901    AGGTGAAGATCCTTTTTGATAATCTCATGACCAAAATCCCTTAACGTGAGTTTTCGTTCC
day5_nocarb     3901    AGGTGAAGATCCTTTTTGATAATCTCATGACCAAAATCCCTTAACGTGAGTTTTCGTTCC

day0            3961    ACTGAGCGTCAGACCCCGTAGAAAAGATCAAAGGATCTTCTTGAGATCCTTTTTTTCTGC
day5_carb       3961    ACTGAGCGTCAGACCCCGTAGAAAAGATCAAAGGATCTTCTTGAGATCCTTTTTTTCTGC
day5_nocarb     3961    ACTGAGCGTCAGACCCCGTAGAAAAGATCAAAGGATCTTCTTGAGATCCTTTTTTTCTGC

day0            4021    GCGTAATCTGCTGCTTGCAAACAAAAAAACCACCGCTACCAGCGGTGGTTTGTTTGCCGG
day5_carb       4021    GCGTAATCTGCTGCTTGCAAACAAAAAAACCACCGCTACCAGCGGTGGTTTGTTTGCCGG
day5_nocarb     4021    GCGTAATCTGCTGCTTGCAAACAAAAAAACCACCGCTACCAGCGGTGGTTTGTTTGCCGG

day0            4081    ATCAAGAGCTACCAACTCTTTTTCCGAAGGTAACTGGCTTCAGCAGAGCGCAGATACCAA
day5_carb       4081    ATCAAGAGCTACCAACTCTTTTTCCGAAGGTAACTGGCTTCAGCAGAGCGCAGATACCAA
day5_nocarb     4081    ATCAAGAGCTACCAACTCTTTTTCCGAAGGTAACTGGCTTCAGCAGAGCGCAGATACCAA

day0            4141    ATACTGTTCTTCTAGTGTAGCCGTAGTTAGGCCACCACTTCAAGAACTCTGTAGCACCGC
day5_carb       4141    ATACTGTTCTTCTAGTGTAGCCGTAGTTAGGCCACCACTTCAAGAACTCTGTAGCACCGC
day5_nocarb     4141    ATACTGTTCTTCTAGTGTAGCCGTAGTTAGGCCACCACTTCAAGAACTCTGTAGCACCGC

day0            4201    CTACATACCTCGCTCTGCTAATCCTGTTACCAGTGGCTGCTGCCAGTGGCGATAAGTCGT
day5_carb       4201    CTACATACCTCGCTCTGCTAATCCTGTTACCAGTGGCTGCTGCCAGTGGCGATAAGTCGT
day5_nocarb     4201    CTACATACCTCGCTCTGCTAATCCTGTTACCAGTGGCTGCTGCCAGTGGCGATAAGTCGT

day0            4261    GTCTTACCGGGTTGGACTCAAGACGATAGTTACCGGATAAGGCGCAGCGGTCGGGCTGAA
day5_carb       4261    GTCTTACCGGGTTGGACTCAAGACGATAGTTACCGGATAAGGCGCAGCGGTCGGGCTGAA
day5_nocarb     4261    GTCTTACCGGGTTGGACTCAAGACGATAGTTACCGGATAAGGCGCAGCGGTCGGGCTGAA

day0            4321    CGGGGGGTTCGTGCACACAGCCCAGCTTGGAGCGAACGACCTACACCGAACTGAGATACC
day5_carb       4321    CGGGGGGTTCGTGCACACAGCCCAGCTTGGAGCGAACGACCTACACCGAACTGAGATACC
day5_nocarb     4321    CGGGGGGTTCGTGCACACAGCCCAGCTTGGAGCGAACGACCTACACCGAACTGAGATACC

day0            4381    TACAGCGTGAGCTATGAGAAAGCGCCACGCTTCCCGAAGGGAGAAAGGCGGACAGGTATC
day5_carb       4381    TACAGCGTGAGCTATGAGAAAGCGCCACGCTTCCCGAAGGGAGAAAGGCGGACAGGTATC
day5_nocarb     4381    TACAGCGTGAGCTATGAGAAAGCGCCACGCTTCCCGAAGGGAGAAAGGCGGACAGGTATC

day0            4441    CGGTAAGCGGCAGGGTCGGAACAGGAGAGCGCACGAGGGAGCTTCCAGGGGGAAACGCCT
day5_carb       4441    CGGTAAGCGGCAGGGTCGGAACAGGAGAGCGCACGAGGGAGCTTCCAGGGGGAAACGCCT
day5_nocarb     4441    CGGTAAGCGGCAGGGTCGGAACAGGAGAGCGCACGAGGGAGCTTCCAGGGGGAAACGCCT

day0            4501    GGTATCTTTATAGTCCTGTCGGGTTTCGCCACCTCTGACTTGAGCGTCGATTTTTGTGAT
day5_carb       4501    GGTATCTTTATAGTCCTGTCGGGTTTCGCCACCTCTGACTTGAGCGTCGATTTTTGTGAT
day5_nocarb     4501    GGTATCTTTATAGTCCTGTCGGGTTTCGCCACCTCTGACTTGAGCGTCGATTTTTGTGAT

day0            4561    GCTCGTCAGGGGGGCGGAGCCTGTGGAAAAACGCCAGCAACGCGGCCTTTTTACGGTTCC
day5_carb       4561    GCTCGTCAGGGGGGCGGAGCCTGTGGAAAAACGCCAGCAACGCGGCCTTTTTACGGTTCC
day5_nocarb     4561    GCTCGTCAGGGGGGCGGAGCCTGTGGAAAAACGCCAGCAACGCGGCCTTTTTACGGTTCC

day0            4621    TGGCCTTTTGCTGGCCTTTTGCTCACATGTTCTTTCCTGCGTTATCCCCTGATTCTGTGG
day5_carb       4621    TGGCCTTTTGCTGGCCTTTTGCTCACATGTTCTTTCCTGCGTTATCCCCTGATTCTGTGG
day5_nocarb     4621    TGGCCTTTTGCTGGCCTTTTGCTCACATGTTCTTTCCTGCGTTATCCCCTGATTCTGTGG

day0            4681    ATAACCGTATTACCGCCTTTGAGTGAGCTGATACCGCTCGCCGCAGCCGAACGACCGAGC
day5_carb       4681    ATAACCGTATTACCGCCTTTGAGTGAGCTGATACCGCTCGCCGCAGCCGAACGACCGAGC
day5_nocarb     4681    ATAACCGTATTACCGCCTTTGAGTGAGCTGATACCGCTCGCCGCAGCCGAACGACCGAGC
```

```
day0            4741    GCAGCGAGTCAGTGAGCGAGGAAGCCTGCAGTTGCAAGACAATATCGACCGCTTGCAGCT
day5_carb       4741    GCAGCGAGTCAGTGAGCGAGGAAGCCTGCAGTTGCAAGACAATATCGACCGCTTGCAGCT
day5_nocarb     4741    GCAGCGAGTCAGTGAGCGAGGAAGCCTGCAGTTGCAAGACAATATCGACCGCTTGCAGCT

day0            4801    TGATAAAGTGATCAAAGAGGGACGAGCAGCTACAGCACTGCTAAGGGAGATCGGAGAGCC
day5_carb       4801    TGATAAAGTGATCAAAGAGGGACGAGCAGCTACAGCACTGCTAAGGGAGATCGGAGAGCC
day5_nocarb     4801    TGATAAAGTGATCAAAGAGGGACGAGCAGCTACAGCACTGCTAAGGGAGATCGGAGAGCC

day0            4861    ACTGCCGCCACCAGTGAAAGTGAAAACAGGTTGGTTTAGTGCTGATTATTACGGCTTTGA
day5_carb       4861    ACTGCCGCCACCAGTGAAAGTGAAAACAGGTTGGTTTAGTGCTGATTATTACGGCTTTGA
day5_nocarb     4861    ACTGCCGCCACCAGTGAAAGTGAAAACAGGTTGGTTTAGTGCTGATTATTACGGCTTTGA

day0            4921    GGACATGCTCACACGTTGGGATAATGATCAATCTAGCCTTTCCATTTATCACAGAGAGAT
day5_carb       4921    GGACATGCTCACACGTTGGGATAATGATCAATCTAGCCTTTCCATTTATCACAGAGAGAT
day5_nocarb     4921    GGACATGCTCACACGTTGGGATAATGATCAATCTAGCCTTTCCATTTATCACAGAGAGAT

day0            4981    AAAAGAGGAGCAACAGGAACAGCAACGAAAACAACAGCAGATAGAAAGAGAAACTAAACA
day5_carb       4981    AAAAGAGGAGCAACAGGAACAGCAACGAAAACAACAGCAGATAGAAAGAGAAACTAAACA
day5_nocarb     4981    AAAAGAGGAGCAACAGGAACAGCAACGAAAACAACAGCAGATAGAAAGAGAAACTAAACA

day0            5041    AAAACAGGAAGATGACAAAAAGAAACGGCAGGACTATGAAAGATATTTATCAAAATACGG
day5_carb       5041    AAAACAGGAAGATGACAAAAAGAAACGGCAGGACTATGAAAGATATTTATCAAAATACGG
day5_nocarb     5041    AAAACAGGAAGATGACAAAAAGAAACGGCAGGACTATGAAAGATATTTATCAAAATACGG

day0            5101    CTATGTCAAAGATGCTGAGTATGACCATATCAGCGACCATGTTGCTTCAGACATTTCCAT
day5_carb       5101    CTATGTCAAAGATGCTGAGTATGACCATATCAGCGACCATGTTGCTTCAGACATTTCCAT
day5_nocarb     5101    CTATGTCAAAGATGCTGAGTATGACCATATCAGCGACCATGTTGCTTCAGACATTTCCAT

day0            5161    GTTTACACGTCTTCAGGCGCAGGCATGGGCAAGTAACGATATGCAAGAGTACATAAGGTG
day5_carb       5161    GTTTACACGTCTTCAGGCGCAGGCATGGGCAAGTAACGATATGCAAGAGTACATAAGGTG
day5_nocarb     5161    GTTTACACGTCTTCAGGCGCAGGCATGGGCAAGTAACGATATGCAAGAGTACATAAGGTG

day0            5221    TTCAAAGCAAAAATACGAGCAGATCAGCGACAGAATAGCCTATGAGCGTGACTTGAAAAA
day5_carb       5221    TTCAAAGCAAAAATACGAGCAGATCAGCGACAGAATAGCCTATGAGCGTGACTTGAAAAA
day5_nocarb     5221    TTCAAAGCAAAAATACGAGCAGATCAGCGACAGAATAGCCTATGAGCGTGACTTGAAAAA

day0            5281    AATCGGGCAATTGAGCCGTATCCTGGATAAAGACAGACAGGCACTTGCTGATATTTTACC
day5_carb       5281    AATCGGGCAATTGAGCCGTATCCTGGATAAAGACAGACAGGCACTTGCTGATATTTTACC
day5_nocarb     5281    AATCGGGCAATTGAGCCGTATCCTGGATAAAGACAGACAGGCACTTGCTGATATTTTACC

day0            5341    GCAGTTGATGCCTTACTATGAGCAGATGCAACAGGCTGTTAATAAACGGAAAATTTTACT
day5_carb       5341    GCAGTTGATGCCTTACTATGAGCAGATGCAACAGGCTGTTAATAAACGGAAAATTTTACT
day5_nocarb     5341    GCAGTTGATGCCTTACTATGAGCAGATGCAACAGGCTGTTAATAAACGGAAAATTTTACT

day0            5401    TGAGAATGAGCGACAGCCATCATTTAAACCACGTTATGACCAAGAACAGCCTAAGCCGAA
day5_carb       5401    TGAGAATGAGCGACAGCCATCATTTAAACCACGTTATGACCAAGAACAGCCTAAGCCGAA
day5_nocarb     5401    TGAGAATGAGCGACAGCCATCATTTAAACCACGTTATGACCAAGAACAGCCTAAGCCGAA

day0            5461    AAAAGACAATGACCTAACTTTTTAATGTGTTACAATAGCATTCAAATGTAACACATTGGA
day5_carb       5461    AAAAGACAATGACCTAACTTTTTAATGTGTTACAATAGCATTCAAATGTAACACATTGGA
day5_nocarb     5461    AAAAGACAATGACCTAACTTTTTAATGTGTTACAATAGCATTCAAATGTAACACATTGGA

day0            5521    GTGAAAAACATGACAGAAGCGACTTTTACCTTTCGGGTAGACCATGACCTAAAGCAAGAA
day5_carb       5521    GTGAAAAACATGACAGAAGCGACTTTTACCTTTCGGGTAGACCATGACCTAAAGCAAGAA
day5_nocarb     5521    GTGAAAAACATGACAGAAGCGACTTTTACCTTTCGGGTAGACCATGACCTAAAGCAAGAA
```

```
day0            5581    TTTTCAAGCCTTGCTAAGACTGTTGATCGGTCGGGGGCGCAGCTAATCCGTGACTTCATG
day5_carb       5581    TTTTCAAGCCTTGCTAAGACTGTTGATCGGTCGGGGGCGCAGCTAATCCGTGACTTCATG
day5_nocarb     5581    TTTTCAAGCCTTGCTAAGACTGTTGATCGGTCGGGGGCGCAGCTAATCCGTGACTTCATG

day0            5641    CGTGACTTTGTTAAGAAGCAACAGGAAGCCGCAGACTATGACAAATGGTTCAAGCAGCAG
day5_carb       5641    CGTGACTTTGTTAAGAAGCAACAGGAAGCCGCAGACTATGACAAATGGTTCAAGCAGCAG
day5_nocarb     5641    CGTGACTTTGTTAAGAAGCAACAGGAAGCCGCAGACTATGACAAATGGTTCAAGCAGCAG

day0            5701    GTACAGATCGGCTTAAATGAAGCCAATGCAGGCAAGTTGATTCCACATGAGGACGTAAAG
day5_carb       5701    GTACAGATCGGCTTAAATGAAGCCAATGCAGGCAAGTTGATTCCACATGAGGACGTAAAG
day5_nocarb     5701    GTACAGATCGGCTTAAATGAAGCCAATGCAGGCAAGTTGATTCCACATGAGGACGTAAAG

day0            5761    GCAGAGTTTGCCGCTAGACGTGCTGCAACATTGGCGAAACTGGCAGCGAAGAATGAAGAT
day5_carb       5761    GCAGAGTTTGCCGCTAGACGTGCTGCAACATTGGCGAAACTGGCAGCGAAGAATGAAGAT
day5_nocarb     5761    GCAGAGTTTGCCGCTAGACGTGCTGCAACATTGGCGAAACTGGCAGCGAAGAATGAAGAT

day0            5821    TGAGTGGACTGAAACAGCTCGCCAAGATCGTAGAAATATCTATGATTATCTTGAAGAACG
day5_carb       5821    TGAGTGGACTGAAACAGCTCGCCAAGATCGTAGAAATATCTATGATTATCTTGAAGAACG
day5_nocarb     5821    TGAGTGGACTGAAACAGCTCGCCAAGATCGTAGAAATATCTATGATTATCTTGAAGAACG

day0            5881    CAACCCTATAGCAGCTATTGAAATTGATGATTTAATTGAAGAAAAGACAGATTTACTTGT
day5_carb       5881    CAACCCTATAGCAGCTATTGAAATTGATGATTTAATTGAAGAAAAGACAGATTTACTTGT
day5_nocarb     5881    CAACCCTATAGCAGCTATTGAAATTGATGATTTAATTGAAGAAAAGACAGATTTACTTGT

day0            5941    TGATAATCGACTGATGGGGCGCACAGGCAGACAGAAAGATACTAGGGAGTTAGTGATACA
day5_carb       5941    TGATAATCGACTGATGGGGCGCACAGGCAGACAGAAAGATACTAGGGAGTTAGTGATACA
day5_nocarb     5941    TGATAATCGACTGATGGGGCGCACAGGCAGACAGAAAGATACTAGGGAGTTAGTGATACA

day0            6001    TCCGCATTATGTGGTTGTATATGACATCACTGATATAATACGGATACTCAGAGTGCTACA
day5_carb       6001    TCCGCATTATGTGGTTGTATATGACATCACTGATATAATACGGATACTCAGAGTGCTACA
day5_nocarb     6001    TCCGCATTATGTGGTTGTATATGACATCACTGATATAATACGGATACTCAGAGTGCTACA

day0            6061    CACATCGCAGGAGTGGTCATGACTTACTCATGTACTTTGGATTATTTAGTGTTATAAAAT
day5_carb       6061    CACATCGCAGGAGTGGTCATGACTTACTCATGTACTTTGGATTATTTAGTGTTATAAAAT
day5_nocarb     6061    CACATCGCAGGAGTGGTCATGACTTACTCATGTACTTTGGATTATTTAGTGTTATAAAAT

day0            6121    CCTGATTTATAAATTTTTTTTGTTAAAAAAGATAAAAGCCCCTTGCAATTGCTTGGGGCT
day5_carb       6121    CCTGATTTATAAATTTTTTTTGTTAAAAAAGATAAAAGCCCCTTGCAATTGCTTGGGGCT
day5_nocarb     6121    CCTGATTTATAAATTTTTTTTGTTAAAAAAGATAAAAGCCCCTTGCAATTGCTTGGGGCT

day0            6181    TTACCGTAATTTATGGGGTACAGATCTTCGATACTGACATATCGGCAATCGAAAGCATTA
day5_carb       6181    TTACCGTAATTTATGGGGTACAGATCTTCGATACTGACATATCGGCAATCGAAAGCATTA
day5_nocarb     6181    TTACCGTAATTTATGGGGTACAGATCTTCGATACTGACATATCGGCAATCGAAAGCATTA

day0            6241    AGGTTTGACGACCGCTAATGATTTCACCACAGGGGCTTAATGTACCTGTCTTAAATTCTA
day5_carb       6241    AGGTTTGACGACCGCTAATGATTTCACCACAGGGGCTTAATGTACCTGTCTTAAATTCTA
day5_nocarb     6241    AGGTTTGACGACCGCTAATGATTTCACCACAGGGGCTTAATGTACCTGTCTTAAATTCTA

day0            6301    AGGTTTTAACTCGCTTTGTCAAGCATAGACCCCAAAAATTTAGCCAATGTCTGTAACTCA
day5_carb       6301    AGGTTTTAACTCGCTTTGTCAAGCATAGACCCCAAAAATTTAGCCAATGTCTGTAACTCA
day5_nocarb     6301    AGGTTTTAACTCGCTTTGTCAAGCATAGACCCCAAAAATTTAGCCAATGTCTGTAACTCA

day0            6361    ATCTGTCCATGTGTGGGTGATGAGGTACAGTGACGCTAGCACACATCGGAAAAACGCTAT
day5_carb       6361    ATCTGTCCATGTGTGGGTGATGAGGTACAGTGACGCTAGCACACATCGGAAAAACGCTAT
day5_nocarb     6361    ATCTGTCCATGTGTGGGTGATGAGGTACAGTGACGCTAGCACACATCGGAAAAACGCTAT

day0            6421    TACTAGGGGAACTGAACAGAGTAGCGGACGCAATGAGTAGTCATTTAATTGGCGGTTATG
```

```
day5_carb          6421    TACTAGGGGAACTGAACAGAGTAGCGGACGCAATGAGTAGTCATTTAATTGGCGGTTATG
day5_nocarb        6421    TACTAGGGGAACTGAACAGAGTAGCGGACGCAATGAGTAGTCATTTAATTGGCGGTTATG

day0               6481    AGCGTGTTCAGGCGGTGCTATCAATCGTAATCATAACAGTGGCAGCTTGATACAGTGATG
day5_carb          6481    AGCGTGTTCAGGCGGTGCTATCAATCGTAATCATAACAGTGGCAGCTTGATACAGTGATG
day5_nocarb        6481    AGCGTGTTCAGGCGGTGCTATCAATCGTAATCATAACAGTGGCAGCTTGATACAGTGATG

day0               6541    TCATCCCTGATGCGAAAGCGACCGACCGACGGTACATCGAATGGGAATACTTTAGGGTGA
day5_carb          6541    TCATCCCTGATGCGAAAGCGACCGACCGACGGTACATCGAATGGGAATACTTTAGGGTGA
day5_nocarb        6541    TCATCCCTGATGCGAAAGCGACCGACCGACGGTACATCGAATGGGAATACTTTAGGGTGA

day0               6601    TTTTTAAGAATCGCTCTAGGGTGAGTATTTCCCATTCAGCTCTGCTCCCTCCCTCTGGTA
day5_carb          6601    TTTTTAAGAATCGCTCTAGGGTGAGTATTTCCCATTCAGCTCTGCTCCCTCCCTCTGGTA
day5_nocarb        6601    TTTTTAAGAATCGCTCTAGGGTGAGTATTTCCCATTCAGCTCTGCTCCCTCCCTCTGGTA

day0               6661    CTTTAATCAAAAGCACTACTAAACATATGTTTTTAAATAAAAAATATTGATATAGAGATA
day5_carb          6661    CTTTAATCAAAAGCACTACTAAACATATGTTTTTAAATAAAAAATATTGATATAGAGATA
day5_nocarb        6661    CTTTAATCAAAAGCACTACTAAACATATGTTTTTAAATAAAAAATATTGATATAGAGATA

day0               6721    ATATTAGTAAGAATAATTAAACAATTGAATATAGATAAATCATTGTTAAATAAAGATTAA
day5_carb          6721    ATATTAGTAAGAATAATTAAACAATTGAATATAGATAAATCATTGTTAAATAAAGATTAA
day5_nocarb        6721    ATATTAGTAAGAATAATTAAACAATTGAATATAGATAAATCATTGTTAAATAAAGATTAA

day0               6781    TTATTAAAATGAATGTATACTTATATATAAATCAATGATTTAAAATATTTGATAAAGAAA
day5_carb          6781    TTATTAAAATGAATGTATACTTATATATAAATCAATGATTTAAAATATTTGATAAAGAAA
day5_nocarb        6781    TTATTAAAATGAATGTATACTTATATATAAATCAATGATTTAAAATATTTGATAAAGAAA

day0               6841    ACTTTTC
day5_carb          6841    ACTTTTC
day5_nocarb        6841    ACTTTTC
```


1. Macguire, A. E., Ching, M. C., Diamond, B. H., Kazakov, A., Novichkov, P. & Godoy, V. G. Activation of phenotypic subpopulations in response to ciprofloxacin treatment in *Acinetobacter baumannii*. *Mol Microbiol* **92**, 138–152 (2014).

2. Ching, C., Gozzi, K., Heinemann, B. & Godoy, V. G. Investigating the regulation of recA in the emerging pathogen *Acinetobacter baumannii*. *The FASEB Journal* **31**, 591.2-591.2 (2017).

3. Schindelin, J., Arganda-Carreras, I., Frise, E., Kaynig, V., Longair, M., Pietzsch, T., Preibisch, S., Rueden, C., Saalfeld, S., Schmid, B., Tinevez, J. Y., White, D. J., Hartenstein, V., Eliceiri, K., Tomancak, P. & Cardona, A. Fiji: an open-source platform for biological-image analysis. *Nature Methods 2012 9:7* **9**, 676–682 (2012).

4. Edgar, R. C. MUSCLE: multiple sequence alignment with high accuracy and high throughput. *Nucleic Acids Res* **32**, 1792–7 (2004).
